# Promising Cathode Materials for Sodium-Ion Batteries from Lab to Application

**DOI:** 10.1021/acscentsci.3c01022

**Published:** 2023-11-13

**Authors:** Shitan Xu, Huanhuan Dong, Dan Yang, Chun Wu, Yu Yao, Xianhong Rui, Shulei Chou, Yan Yu

**Affiliations:** † School of Materials and Energy, Guangdong University of Technology, Guangzhou, Guangdong 510006, China; ‡ Institute for Carbon Neutralization, College of Chemistry and Materials Engineering, Wenzhou University, Wenzhou, Zhejiang 325035, China; § Wenzhou Key Laboratory of Sodium-Ion Batteries, Wenzhou University Technology Innovation Institute for Carbon Neutralization, Wenzhou, Zhejiang 325035, China; ∥ Hefei National Research Center for Physical Sciences at the Microscale, Department of Materials Science and Engineering, CAS Key Laboratory of Materials for Energy Conversion, University of Science and Technology of China, Hefei, Anhui 230026, China

## Abstract

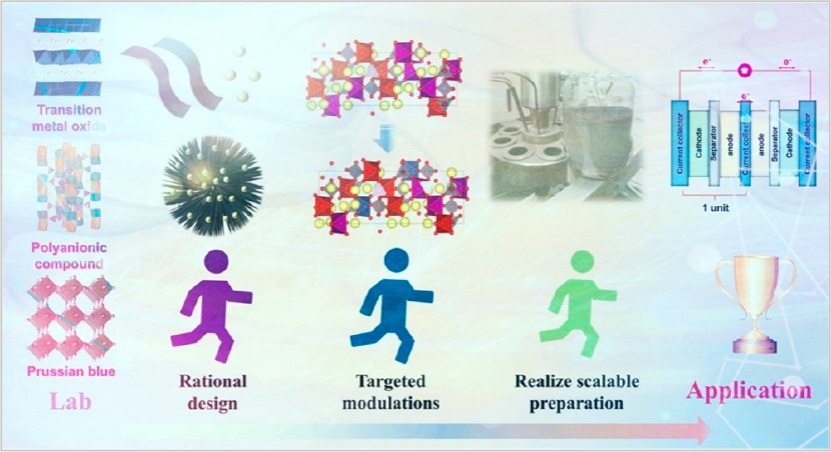

Sodium-ion batteries (SIBs) are seen as an emerging force for future large-scale energy storage due to their cost-effective nature and high safety. Compared with lithium-ion batteries (LIBs), the energy density of SIBs is insufficient at present. Thus, the development of high-energy SIBs for realizing large-scale energy storage is extremely vital. The key factor determining the energy density in SIBs is the selection of cathodic materials, and the mainstream cathodic materials nowadays include transition metal oxides, polyanionic compounds, and Prussian blue analogs (PBAs). The cathodic materials would greatly improve after targeted modulations that eliminate their shortcomings and step from the laboratory to practical applications. Before that, some remaining challenges in the application of cathode materials for large-scale energy storage SIBs need to be addressed, which are summarized at the end of this Outlook.

## Abstract 2

The reasonable structural design and scalable preparation of cathode materials are crucial factors for the successful transition of sodium-ion batteries from the lab to industry.

## 1 Introduction

Sodium-ion batteries (SIBs) offer safer and more environmentally sustainable solutions to lithium-ion batteries (LIBs) with comparable performance.^1^ Despite great potential in applications for high-power energy storage systems, current SIBs still suffer from drawbacks, such as an inferior charge and discharge rate (low power density), lower specific capacity (low energy density), and short cycle life.^2−4^ Cathode materials play a central role in determining the electrochemical performance of SIBs. However, the current SIB cathodes face challenging issues, including undesirable phase changes along cycling, sluggish Na ion mobility, and unfavorable interphase formation between electrode and electrolytes, which are mainly associated with the larger Na ions compared to Li ions.^5,6^ Hence, the selection of electrode materials, especially cathode materials featuring high energy densities and prolonged cycle life, that could buffer the repeated Na^+^ (de)insertion is quite crucial.

**Figure 1:**
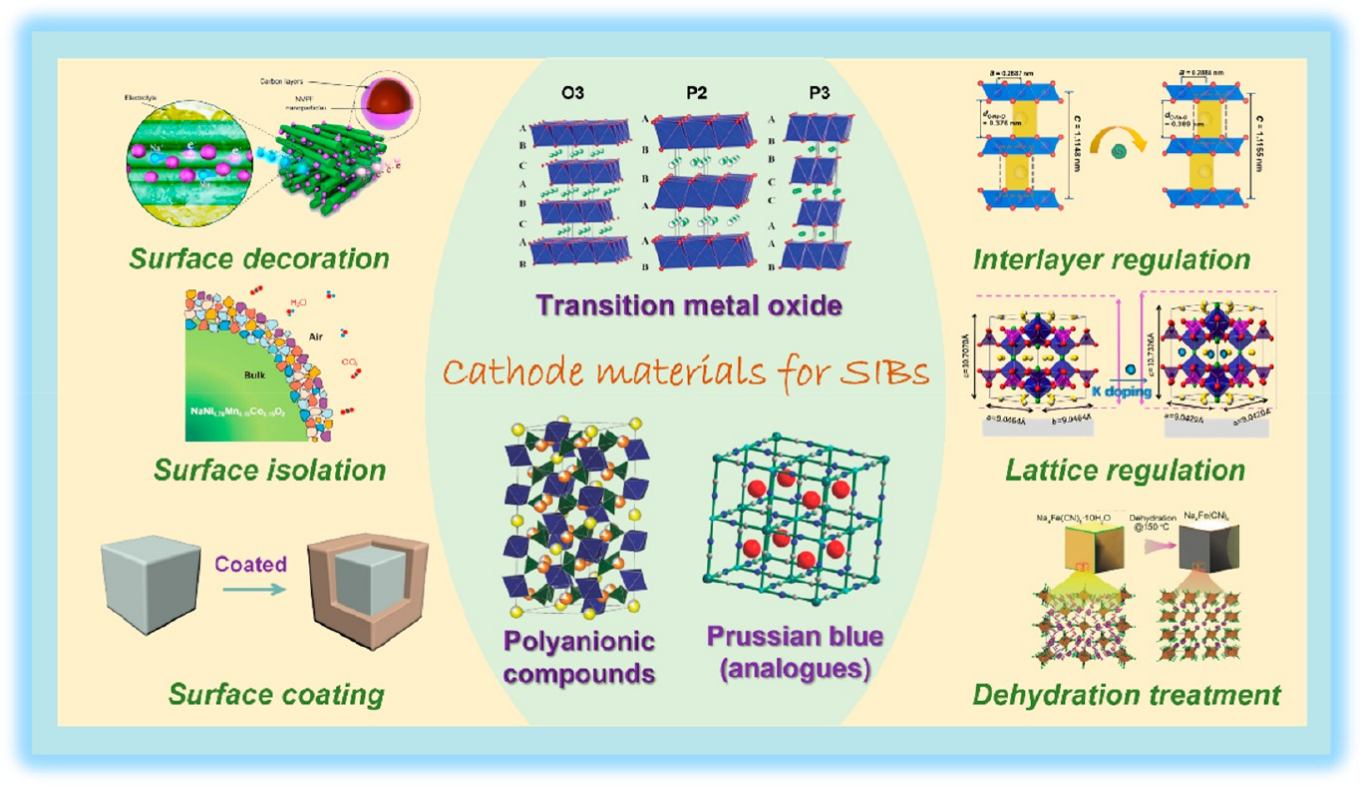
Overview of cathodic materials and their effective modification strategies. Top left image reproduced with permission from ref (182). Copyright 2016 American Chemical Society. Middle left image reproduced with permission from ref (44). Copyright 2018 American Chemical Society. Bottom left image reproduced with permission from ref (138). Copyright Wiley-VCH. Top center image reproduced from (6). Copyright 2020 Wiley-VCH. Bottom-left center image reproduced with permission from ref (12). Copyright 2018 Wiley-VCH. Bottom-right center image reproduced with permission from ref (9). Copyright 2018 Wiley-VCH. Top right image reproduced with permission from ref (68). Copyright 2022 Springer Nature. Middle right image reproduced with permission from ref (183). Copyright 2019 Elsevier. Bottom right image reproduced with permission from ref (184). Copyright 2016 Wiley-VCH.

Up to now, three categories of materials have been explored as cathodic alternatives for SIBs: transition metal oxides, polyanionic compounds, and Prussian blue analogs (PBAs). Each category of the cathode materials has their own features and inherent problems. The transition layered oxides with large spacers for Na^+^ storage have high reversible specific capacities, high energy densities, and excellent rate capabilities combined with susceptibly convertible technologies. However, such a layered structure is prone to collapse when accommodating large-radius Na^+^ for (de)insertion, resulting in an unsatisfactory cycle lifespan; besides, most layered oxides are sensitive to the moisture in the air and the absorbent, thus bringing about storage difficulties.^3^,^7^^8^ Polyanionic compounds possess high working voltages and excellent thermal/cyclic stability but suffer from inferior intrinsic electronic conductivity that causes a low specific capacity and poor rate capability.^9−11^ Prussian blue and its analogs have the advantages of low cost, great rate performance, and adjustable working voltage, but the stubborn lattice water is difficult to remove and reduces the chemical stability and structural stability of the PBA material.^12^ Effective improvement strategies have been proposed to address the shortcomings of different cathode materials, such as surface modification (isolation or coating), structural design, and lattice or interlayer modulation, in order to realize the high energy density, superior rate capability, and long service lifespan of SIBs (Figure 1).

In this Outlook, we summarized the recent progress of the major cathodic materials for SIBs, introducing their crystal structures, physicochemical properties, and electrochemical applications. Finally, the remaining challenges in the application of these cathode materials for future large-scale energy storage SIBs are discussed. We hope this Outlook can make a guiding contribution to the development of cathode materials for high-energy SIBs.

## 2 Transition Metal Oxides

**Figure 2:**
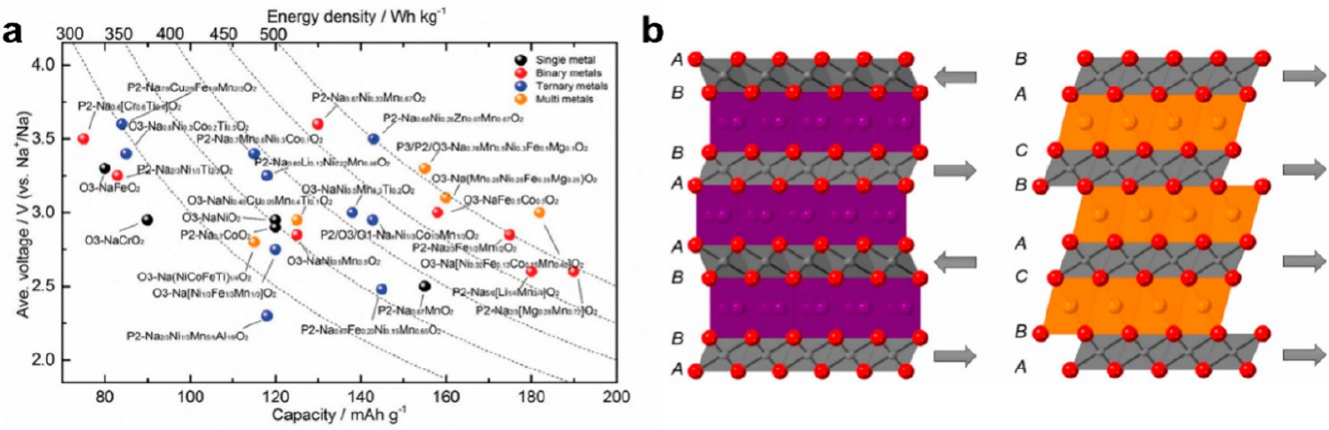
(a) Summary of the electrochemical properties of various layered oxide cathodes. (b) Illustration of P2 (left) and O3 (right) crystal structures. Reproduced with permission from from ref (20). Copyright 2021 IOP.

The composition and structure of current cathodes for SIBs are mostly inherited from the LIB cathode analogs, while it is observed that the insertion/alloying of larger Na ions into/with the electrodes leads to distinctive crystal structures, in other words, offering increased structural versatility. In general, the materials that have been investigated as cathodes for SIBs include layered- and tunnel-structured transition metal oxides, polyanion compounds, and Prussian blue analogs (PBAs). Among them, layered transition metal oxides are considered as the most important cathodic alternatives for SIBs. Figure 2a compares the voltages, capacities, and energy densities of the layered metal oxide cathodes composed of single, binary, ternary and multicomponent metal ions. Typical Na-based layered transition oxides, i.e., NaMO_2_ (M = Ni, Co, Mn, Fe, Cr, V, etc.), exist in different crystal structures denoted as P2, P3, O2, and O3 according to Delmas’ notation.^13^ O and P indicate the coordination environment of Na^+^, in which O represents the Na occupancy at the octahedron sites surrounded by six oxygens and P represents the Na occupancy at the center of prism sites surrounded by six oxygens. Among them, O3-phase and P2-phase are most widely investigated as cathodes for SIBs, and their crystal structures are illustrated in Figure 2b. Typical examples of O3-type metal oxides include NaFeO_2_,^14^ NaNiO_2_,^15^ and NaNi_1/2_Mn_1/2_O_2_,^16^ and those for P2-type metal oxides include Na_2/3_MnO_2_,^17^ Na_0.7_CoO_2_,^18^ and Na_2/3_Ni_1/3_Mn_2/3_O_2._^19^ Generally, the P2 structure renders the best power performance for SIBs, as Na ion diffuse through rectangular faces between adjacent trigonal prismatic environments, which is unavailable in LIBs. The O3-structured materials are outstanding in capacity, as they have the highest sodium stoichiometries. In recent years, hybrid P2/O3-structured materials have attracted extraordinary attention for simultaneous optimization of the power and energy density for SIBs.

### 2.1 Na-Free Transition Metal Oxides

Vanadium oxides have been investigated as the cathodic materials for SIBs due to their features of high capacity and low cost. VO_2_(A) is unstable during electrochemical reactions,^21^ but VO_2_(B) is considered a more suitable cathode for SIBs because its layered structure allows for rapid Na ion diffusion; the corresponding theoretical capacity is as high as 322 mA h g^–1^, which is associated with one e^–^ transfer during the (de)sodification process.^22,23^ However, VO_2_(B) is metastable and less conductive, so rapid capacity fading was often observed for VO_2_(B) because of the drastic volume expansion, dissolution, and aggregation of the electrode material. In comparison, V_2_O_5_ shows higher chemical and thermal stability with layered and orthorhombic structures, both of which are electrochemically active. The theoretical capacity of V_2_O_5_ varies depending on the number of transfer electrons involved in the redox reaction (capacities of 294 and 441 mA h g^–1^ corresponding to 2e^–^ and 3e^–^ participating in the reaction, respectively). The electrochemical process of V_2_O_5_, however, starts to degrade with the morphology change or crystal structure collapse upon cycling, which is also known as “lattice breathing”.^24^ The stability of V_2_O_5_ electrodes can be enhanced by inserting larger cations (Na^+^, NH^4+^) or water molecules into the crystal interlayers.^25,26,27^ Constructing V_2_O_5_ aerogels with highly porous 3D networks has also proved to be an efficient strategy for enhancing their performance.^28^ Apart from the crystallized structure, the amorphous V_2_O_5_ also demonstrates a Na storage property.^29^ Furthermore, manganese oxides, such as α-MnO_2_ and β-MnO_2_, show promise as cathodes for SIBs, and they exhibit a theoretical Na^+^ storage capability of 308 mA h g^–1^.^30^ In particular, the compact tunnels in β-MnO_2_ along the [001] direction are favorable for the Na^+^ insertion/extraction and contribute to the better Na storage capacity. Introduction of exchangeable guest cations into the MnO_2_ framework can modulate the Na storage performance, though the exact role and effects of the guest ions still needs further investigation.^31^

### 2.2 Layered Sodium Monometallic Oxides

The sodium monometallic oxides often suffer from poor stability and rapid degradation due to the continuous phase changes of the oxides, especially at high voltages. For example, α-NaFeO_2_ is a O3-type cathode material with excellent thermal stability with an active Fe^3+^/Fe^4+^ redox couple. Under higher voltages, their electrochemical performance degrades mainly due to the Jahn–Teller distortion and polarization. Fe^4+^ is reduced to Fe^3+^ at the charged state, and the excessive Fe^3+^ will migrate and block the diffusion pathways of Na ions, causing the degradation of performance.^32^ A recent finding also points out both oxygen reactivity and the Fe^3+^/Fe^4+^ contribute to the electrochemical activity of NaFeO_2_, evidenced by the diminished Fe^3+^ ions under high voltages.^33^ Partially substituting Fe with Ni^3+^ ions or Co^3+^ ions, reducing the particle sizes, or surface modification can help enhance the stability of NaFeO_2_.^34^ Na_*x*_CoO_2_ exists in both the P2 and O3 phase, where the P2 phase is relatively more stable without gliding of the CoO_6_ slabs during the charge and discharge process.^35,36^ The stability of this material is mainly affected by the Na^+^/vacancy ordering, which can be relieved by partial substitution of the Co ions with Ni^3+^, Mn^2+^, and Ti^4+^.^36,37,38^ Similar to MnO_2_, Na_*x*_MnO_2_ suffers from severe volume change induced by the Jahn–Teller distortion and dissolution of Mn species during the electrochemical reactions.^39,40^ The disproportion of Mn^3+^ into Mn^4+^ and Mn^2+^ results in the dissolution of Mn species into the electrolyte. High-temperature quenching can remove the Mn vacancies and suppress their dissolution, while also creates more Mn^3+^ and leads to more severe Jahn–Teller distortion.^17^ An alternative strategy is to quench the electrode using liquid N_2_ which can eliminate the Mn vacancies without creating extra Mn^3+^ ions.^41^ Although Ni has been widely used as a doping metal, Na_*x*_NiO_2_ electrodes show inferior performance and poor stability when used as cathodes for SIBs. Decay of a high-Ni cathode is mainly associated with the insertion of water and carbonate ions between the TMO_2_ slabs and oxidation of the electrodes.^42−44^ Washing with ethanol, reducing interlayer spacing, and using proper electrolyte are effective strategies to enhance their stability.^45^ NaVO_2_ shows a similar structure to O3 a-NaFeO_2_, while the pure-phase NaVO_2_ is difficult to synthesize. NaVO_2_ can only be reversibly cycled in the narrow working window of 1.4–2.5 V.^46^ When a higher voltage was applied, the composition underwent continuous variation with the emergence of many potential plateaus. NaCrO_2_ has a theoretical capacity of ∼250 mA h g^–1^, but it faces a similar issue of poor irreversibility at high voltages, just like NaVO_2_.^47^ It has been reported that partial substitution of Cr by Ru and Ca ions can be effective in obtaining a more stable NaCrO_2_ electrode.^48,49^ The Ru substitution can possibly improve the working plateau (presenting an extra high voltage plateau at 3.8 V) and shows an excellent cycling performance (80.7% capacity retention after 1100 cycles). When Ca is doped in NaCrO_2_, it can improve the cycling performance (76% for 500 cycles) and air stability (slight change observed after exposure for a month). For layered sodium monometallic oxides, element doping is primarily applied to restrict the influence of Na^+^/vacancy ordering and the Jahn–Teller effect in Na_*x*_MO_2_ (M = Fe, Co, Mn, Ni, etc.), as well as to improve the structural stability (air-stability). In this regard, some inactive elements such as Ti, Ru, and Ca have shown effective results in improving the aforementioned effects.

**Figure 3:**
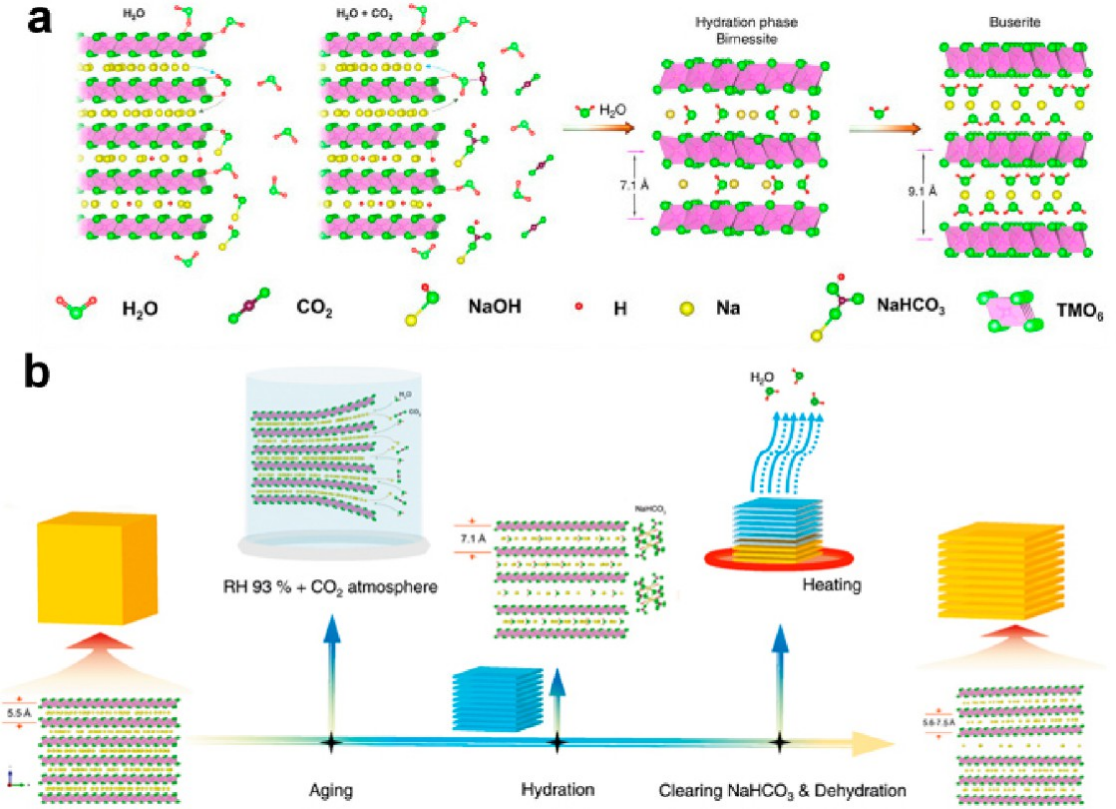
(a) Schematic illustration of the hydration and CO_2_ uptake process when exposing layered metal oxides in air. Reproduced with permission from ref (51). Copyright 2023 American Chemical Society. (b) Water-mediated synthetic process that can expand the interlayer spacing between the Na^+^ layers. Reproduced with permission from ref (54). Copyright 2021 Springer Nature.

Another challenge with the layered metal oxides is their hygroscopic nature, as they tend to uptake water and CO_2_ from air, which results in fast capacity decay and dissolution of the electrodes (as illustrated in Figure 3a).^42,50,51^ Surface modification by coating ZrO_2_, Na_2_TiO_7_, or AlF_3_ can be effective in promoting ion diffusion, enhancing air stability, and preventing infiltration of the electrolyte, therefore improving the stability of the cathode materials.^44,52,53^ The hygroscopic nature of Na_*x*_MnO_2_ has been utilized to expand the interlayer spacing between Na layers, which can facilitate the Na ion transport and suppress the phase transformations during the electrochemical reactions. As illustrated in Figure 3b, the continuous aging and hydration process allows full uptake and insertion of CO_2_ and water molecules, leading to significantly enlarged Na^+^ layer spacing in the P2–Na_0.67_MnO_2_.^54^

### 2.3 Layered Sodium Multimetallic Oxides

Nowadays, most of the research attention has been devoted to the development of multimetallic oxide cathodes for high-performance SIBs. With the cooperative benefits from different metal ions, the common issues encountered by the monometallic oxide cathodes, such as the Jahn–Teller distortion, undesired Na^+^/vacancy ordering, and structural instability, can be effectively addressed. Substituting Mn ions with Cu^2+^ ions can modulate the air and water sensitivity of Na_*x*_MnO_2_.^55^ Partial substitution of Cr^3+^ with Ti^4+^ in Na_2/3–*x*_Cr_2/3_Ti_1/3_O_2_ endowed the electrode with a higher operating voltage.^56^ The presence of Ru in the Na_0.88_Cr_0.88_Ru_0.12_O_2_ suppressed the irreversible migration of Cr ions and elevated the operating voltage.^48^ In particular, forming a O3/P2 hybrid structure has become a popular strategy to achieve cathode materials with both high capacity and high stability. Typical O3/P2 hybrids include the O3-type NaNi_0.5_Mn_0.5_O_2_ mixed with minor P2 phases, such as Na_0.66_Li_0.18_Mn_0.71_Ni_0.21_Co_0.08_O_2_,^57^ Na_0.67_Mn_0.55_Ni_0.25_Ti_0.2–*x*_Li_*x*_O_2_,^58^ and Na_*x*_[Ni_0.2_Fe_*x*–0.4_Mn_1.2–*x*_]O_2_ (*x* = 0.7–1.0).^59^ These hybrid-structured electrodes generally showed smoother charge–discharge profiles, reduced polarizations, and higher capacities during the cycling process.

Metal substitution is effective in suppressing the phase transition during the reaction process. For example, Al and Fe substitution can suppress the undesired phase transition in Na_0.67_Al_0.1_Fe_0.05_Mn_0.9_O_2._^41^ The Jahn–Teller distortion in Na_*x*_MnO_2_ can be suppressed by the introduction of Li^+^, Mg^2+^, Fe^3+^, Ni^3+^, and Ti^4+^ ions.,^60,61^ The reason for Li^+^ and Mg^2+^ substitution is that they can oxidize Mn^3+^ into Mn^4+^ and thus reduce the Jahn–Teller distortion.^62,63^ Besides cation doping, doping with fluorine has lowered the energy barrier for Na ion diffusion in Na_0.46_Mn_0.93_Al_0.07_O_1.79_F_0.21_.^64^ A honeycomb-ordered O3–Na_3_Ni_2_Sb_6_O_6_ has demonstrated a high capacity and stability upon cycling.^65^ The enhanced stability originated from the presence of the honeycomb-ordered Ni_2_SbO_6_ slabs. Substituting 1/3 of Ni with Sb led to the formation of the Ni_6_-ring structure inside NaNiO_2_, which degenerated the electronic orbitals and increased the redox potential of the cathode. Partial substituting Ni^2+^ with inactive cations such as Zn^2+^, Mg^2+^, and Ca^2+^ resulted in the formation of the nanodomains composed of intergrown P3–O1 phases within the crystal structure of Na_0.2_Ni_0.45_Zn_0.05_Mn_0.4_Ti_0.1_O_2_, which not only fully strengthened the potential capacity of the metal oxide electrode but also suppressed the undesired phase transition and structural degradation upon cycling.^66^ Potassium ions were introduced into the P2–K_0.5_Mn_0.7_Fe_0.2_Ti_0.1_O_2_ and served as pillar ions to expand the lattice for Na ion insertion and deinsertion and stabilize the crystal structure.^67^

**Figure 4:**
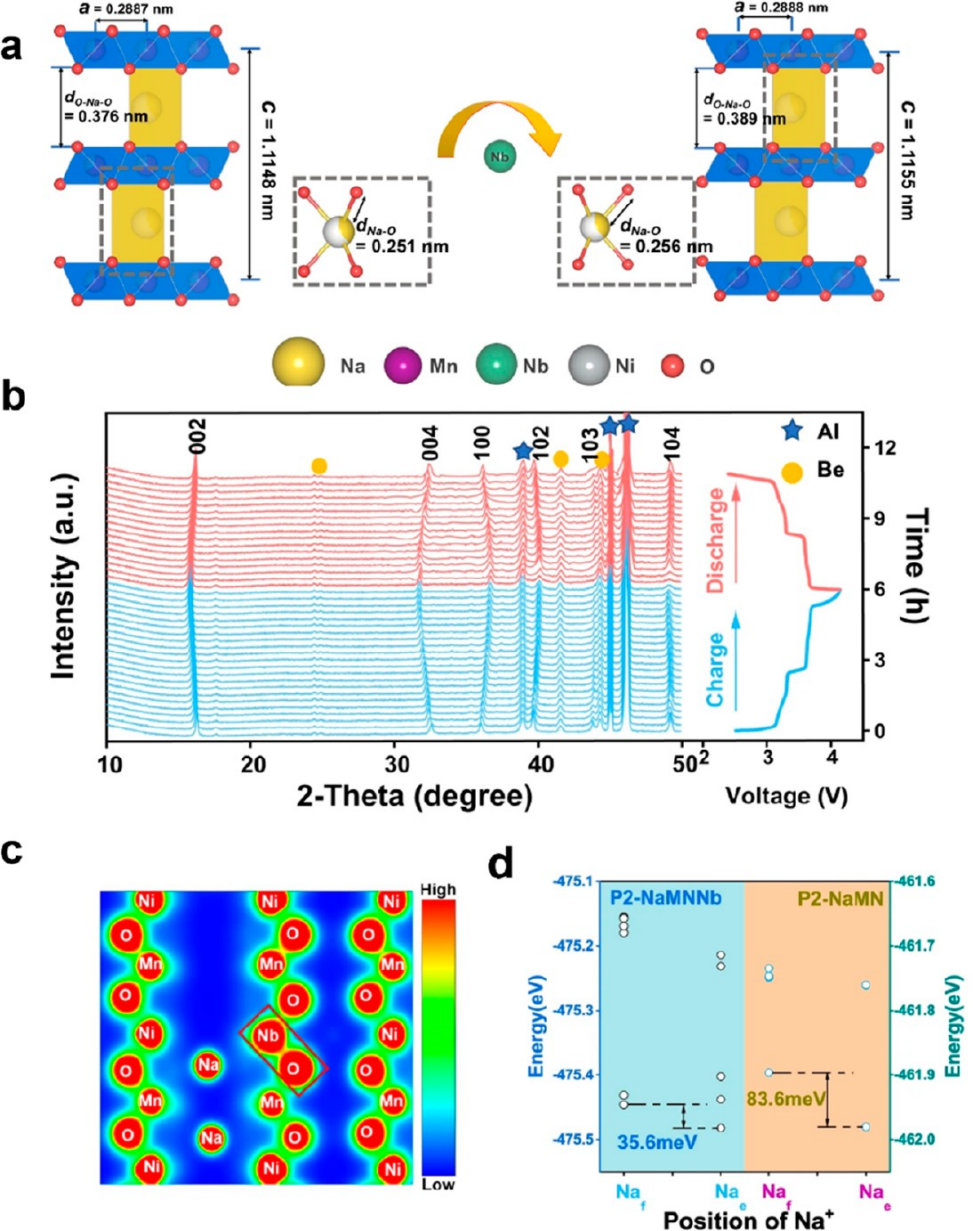
(a) Variation of the crystal structures in Na_2/3_Mn_2/3_Ni_1/3_O_2_ with Nb doping. (b) In situ XRD patterns of the electrodes during the charge and discharge process. (c) Charge density distribution in the Nb-doped Na_2/3_Mn_2/3_Ni_1/3_O_2._. (d) Calculated energy difference between different Na sites with and without Nb doping. Reproduced with permission from ref (68). Copyright 2022 from Springer Nature.

To address the irreversible structural changes or phase transitions of P2–Na_2/3_Mn_2/3_Ni_1/3_O_2_ aroused by severe interfacial transition and metal dissolution, Nb-doped P2–Na_0.78_Ni_0.31_Mn_0.67_Nb_0.02_O_2_ with proper surface modifications enabled fast Na^+^ (de)intercalation for efficient battery cycling even at low temperatures such as −40 °C, showing a high specific capacities of 83.6 and 62.9 mA h g ^–1^ at 920 and 1.84 A g^–1^, respectively. Besides, superior long-term cyclability at low temperatures is demonstrated by the high capacity retention of 76% at 368 mA g^–1^ over 1800 cycles.^68^ As shown in the refined crystal structure in Figure 4a, Nb doping can expand the spacing between the TM layers from 0.376 to 0.389 nm and extend the Na–O bond from 0.251 to 0.256 nm, endowing Na^+^ with enhanced (de)intercalation capabilities. The in situ X-ray diffraction (XRD) spectra shown in Figure 4b illustrate that all the characteristic diffraction peaks revert to their original initial positions without the appearance of any new phase after a charge/discharge cycle. The charge density distribution of the Nb-doped Na_2/3_Mn_2/3_Ni_1/3_O_2_ reflects that the interaction between TM and O is more intense than that between Na and O (Figure 4c), and the energy calculation implies that the Na hopping is easier when Nb is doped in (Figure 4d).

### 2.4 Novel Metal Oxide Cathodes

**Figure 5:**
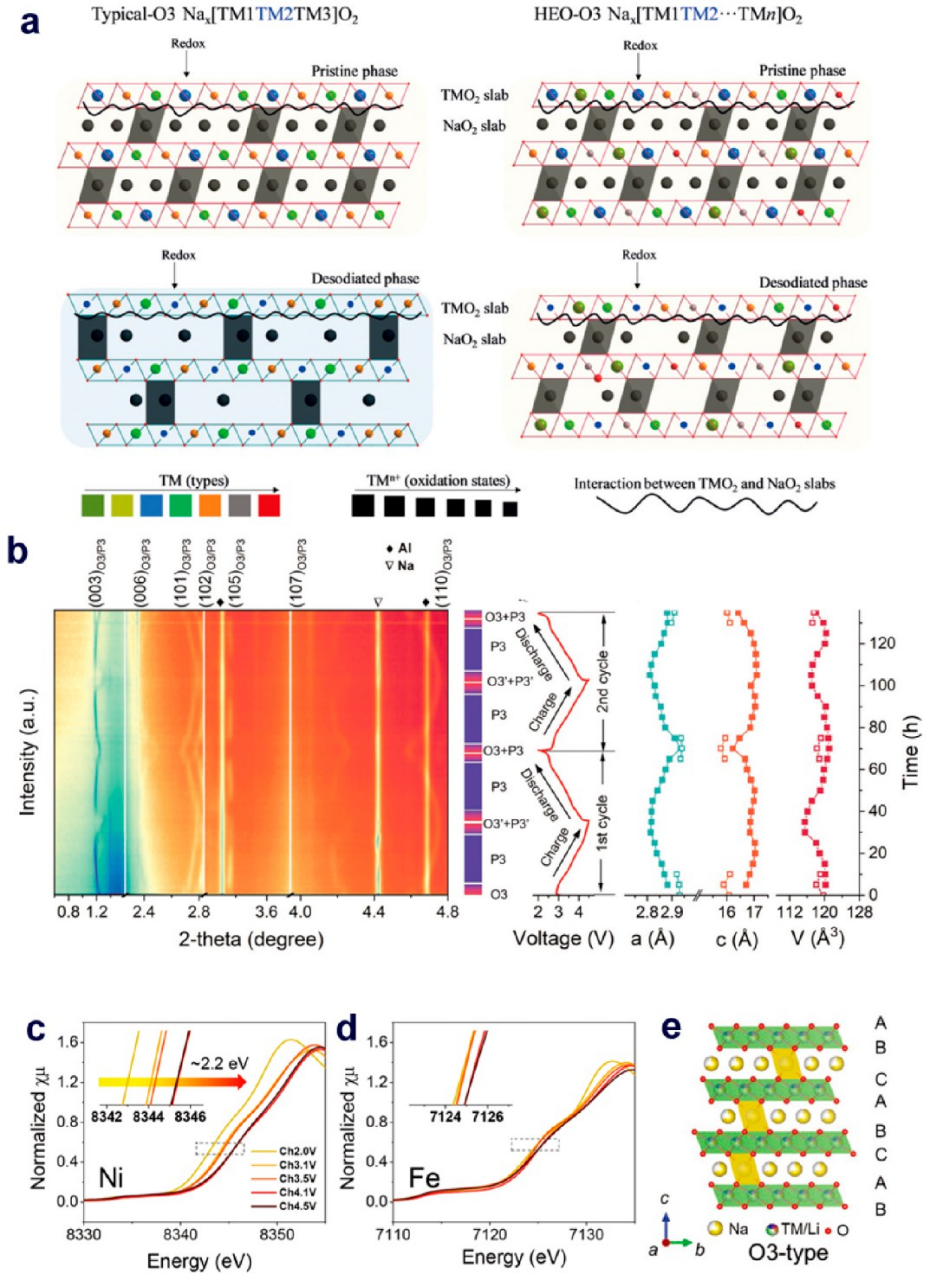
(a) Illustration of the mechanisms of traditional metal oxides and multicomponent HEO in stabilizing the O3-type structure. Reproduced with permission from ref (70). Copyright 2019 Wiley-VCH. (b) In situ high-energy XRD patterns during the first charge–discharge cycles of Na_2/3_Li_1/6_Fe_1/6_Co_1/6_Ni_1/6_Mn_1/3_O_2_ cathode. XANES spectra of the (c) Ni K edge and (d) Fe K edge. (e) O3-structure with a superlattice. Reproduced with permission from ref (71). Copyright 2022 Wiley-VCH.

Apart from the conventional layered metal oxides cathodes, metal oxides cathodes that adopt novel compositions and crystal structures or employ a novel Na storage mechanism has also been explored. Middle-entropy oxides (MEOs) and high-entropy oxides (HEOs) are novel categories of multimetallic single-phase solid solution oxides with multiple metals sharing the crystallographic sites and stabilizing the host structure through the “entropy-stabilization effect”.^69^ In addition, the oxygen vacancies generated among the metal ions can effectively promote the Na ion diffusion. For example, the multicomponent in O3-type NaNi_0.12_Cu_0.12_Mg_0.12_Fe_0.15_Co_0.15_Mn_0.1_Ti_0.1_Sn_0.1_Sb_0.04_O_2_ results in different local interactions between elements in TMO_2_ slabs and Na in NaO_2_ slabs and achieves entropy stabilization on the host (illustrated in Figure 5a),^70^ which has suppressed the phase transition and benefited the long-term cycling of the high-entropy metal oxides electrodes.

A high-entropy Na_2/3_Li_1/6_Fe_1/6_Co_1/6_Ni_1/6_Mn_1/3_O_2_ cathode with a superlattice structure with Li/transition metal ordering presented excellent electrochemical performance.^71^ The as-prepared cathode shows high reverse capacities of 172.3 mA h g^–1^ in the first cycle at 0.1 C and 78.2 mA h g^–1^ at 10 C, demonstrating its superior rate capacity. Excellent cycling stability with the retention of 63.7% after 300 cycles at a current density of 5 C was also validated. In situ high-energy XRD confirmed the O3-type structure of the original cathode, which underwent a fast O3–P3 phase transition at the initial stage of charging (Figure 5b). X-ray absorption spectroscopy (XAS) analysis (Figure 5c, d) reveals that the Ni^2+^/Ni^3+^/Ni^4+^ and Fe^3+^/Fe^4+^ redox couples jointly contributed to the high reversible capacity, while Co doping enhanced the electronic conductivity. Moreover, the superlattice structure of the electrode maintained stable even after long cycles, as illustrated in Figure 5e.

Another emerging type of cathode material is metal oxides involving anionic redox reactions (ARRs) for ion storage. Some of the metal oxides are anionic redox-active intrinsically. For example, NaVO_3_ and Na_3_RuO_4_ are intrinsic ARR materials with both cathodic and anionic redox couples that contribute to the high capacity.^72,73^ With proper engineering over the structure and composition, ARR electrodes can be created through metal ion doping. The Zn-doped P2–Na_2/3_Mn_1–*y*_Zn_*y*_O_2_ electrode showed high oxygen redox activity associated with nonbonding O(2p) orbitals.^74^ Doping metal ions causes ionic bonding, such that the electrons fully localized on the oxygen anions and the TM deficiency were the key to activate the oxygen anion redox activity. Metal ions that can form ionic bonds with oxygen, e.g., Li–O, Na–O, and Mg–O bonds, can be doped to create O(2p) nonbonding orbitals so that the electrons are fully localized on the oxygen anions. On the other hand, the P2 structure in ARR Na_0.72_[Li_0.24_Mn_0.76_]O_2_ could be stabilized even when 0.93 Na was extracted.^75^ The change in the oxygen radii and charges carried by the oxygen ions resulted in a decrease in oxygen repulsion around the empty Na layer and hence stabilized the structure. Besides, with the double redox reaction from both Ni^2+^/Ni^4+^ and O^2–^/O^*n*–^, the higher redox potential of Na[Mn_0.5_Ni_0.5_]O_2_ compared to that of NaMnO_2_ with a single redox reaction was expected.^76^ However, it was also noticed that the ARR electrodes suffer from the structural degradation in complex phase transitions and loss of oxygen during the cycling process.

### 2.5 Scalable Preparation of Transition Metal Oxides

The reports on the scalable preparation of transition metal oxides mainly center on the coprecipitation method. It has the ability to achieve layered oxide cathode materials with a smooth surface, uniform particle size distribution, and high compaction density by controlling reaction conditions, making this method more suitable for industrial production. Sun and coworkers proposed a nickel-rich Na(Ni_0.65_Co_0.08_Mn_0.27_)O_2_ material with a core–shell structure, which was prepared through coprecipitation followed by milling at a rotational speed of 1000 r min^–1^ at 50 °C.^52^ Its first discharge specific capacity was 168 mA h g^–1^ measured at 0.5 C within the voltage range of 1.5–4.0 V, and the capacity retention rate after 50 cycles was found to be 77%. Ding et al. synthesized a novel Ni-rich O3-type Na[Ni_0.60_Fe_0.25_Mn_0.15_]O_2_ cathode for SIBs via the industrially feasible hydroxide coprecipitation method followed by high-temperature calcination.^77^ By reducing the charge voltage from 4.2 to 4.0 V (i.e., eliminating the high-voltage O3″ phase), the electrode exhibited an excellent overall performance, including the high reversible capacity of 152 mA h g^–1^ and a superior capacity retention of ∼84% after 200 cycles at 0.5 C.

## 3 Polyanionic Compounds

Among varous cathode materials, the polyanionic-type cathodes also attract much attention due to their high working potential and great structural stability. The general formula of polyanionic-type cathode materials in SIBs is NaM_*x*_(XO_*y*_)_*z*_·*n*H_2_O, where M represents a transition mental element, such as V, Fe, Mn, Cr, Ni, Ti, etc., and X is nonmetal element like P, S, Si, As, Mo, or W.^9,10^ According to the different type of polyanion, polyanionic cathode can be divided into the following categories: phosphate, sulfate, silicate, borate, and mixed-polyanion materials. The high induction effect brought by the polyanionic XO_4_ can effectively increase the working voltage of the cathode, and the polyhedral connection of XO_4_ and MO_6_ makes the structure stable, which can withstand repeatedly Na^+^ (de)insertion, prolonging the working life of the batteries.^78^ Nevertheless, polyanionic materials also face certain problems, including intrinsically inferior electronic conductivity, causing lower specific capacity and poor rate performance. In order to solve these problems, it is essential to have a comprehensive understanding of polyanionic materials, ranging from the crystal structures, their basic physicochemical properties, the Na storage mechanism, and current advances of polyanionic cathodes for SIBs.

### 3.1 Characteristics of Polyanionic Compounds

#### 3.1.1 Phosphates

**Figure 6:**
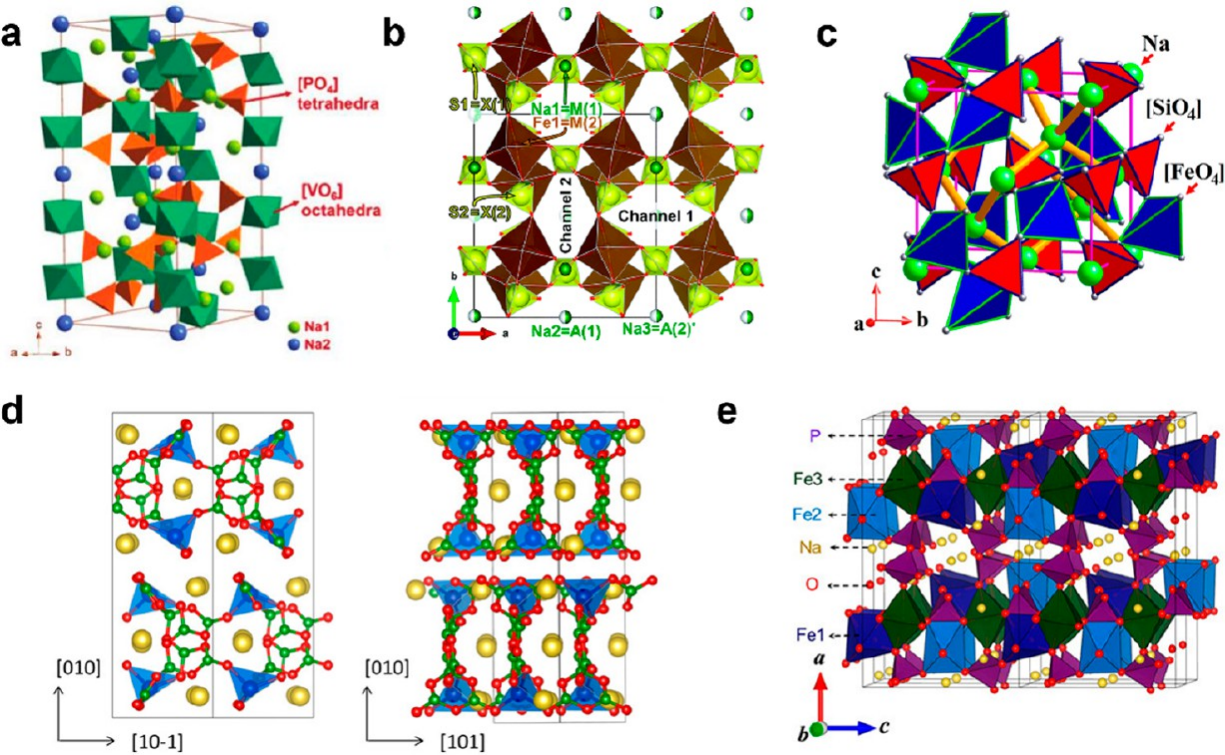
(a) Crystal structure of Na_3_V_2_(PO_4_)_3_. Reproduced with permission from ref (87). Copyright 2019 Royal Society of Chemistry. (b) Crystal structure of Na_2_Fe_2_(SO_4_)_3_. Reproduced with permission from ref (88). Copyright 2019 Royal Society of Chemistry. (c) Crystal structure of Na_2_FeSiO_4_. Reproduced with permission from ref (82). Copyright 2016 American Chemical Society. (d) Crystal structure of Na_3_FeB_5_O_10_. Reproduced with permission from ref (83). Copyright 2016 American Chemical Society. (e) Crystal structure of Na_4_Fe_3_(PO_4_)_2_(P_2_O_7_). Reproduced with permission from ref (89). Copyright 2012 American Chemical Society.

The phosphate-based materials in SIBs can be divided to three categories: orthophosphate NaMPO_4_ (M = Fe, Mn, Ni), NASICON-type Na_*x*_M_*y*_(PO_4_)_3_ (M = V, Fe, Mn, Ti), and pyrophosphate Na_2_MP_2_O_7_ (M = V, Fe, Co, Mn).^79^ As a representative of NaMPO_4_, the crystal structures of NaFePO_4_ are mainly olivine type (o-NaFePO_4_) and maricite type (m-NaFePO_4_). o-NaFePO_4_ consists of FeO_6_ octahedra and PO_4_ tetrahedra forming a spatial skeleton, with Na^+^ occupying the cosided octahedra and forming a long chain along the *b*-axis direction (its theoretical capacity is 156 mA h g^–1^ based on 2e^–^ transportation); in contrast, the positions of Na^+^ and Fe^2+^ in m-NaFePO_4_ are reversed and the position of PO_4_^3–^ remains unchanged, blocking the Na^+^ diffusion channel and resulting in poor or even inactive electrochemical performance. With the structural stability of m-NaFePO_4_, methods for stimulating its electrochemical activity are being continuously studied. As shown in Figure 6a, the classical NASICON-type Na_3_V_2_(PO_4_)_3_ belonging to Na_*x*_M_*y*_(PO_4_)_3_ (M = V, Fe, Mn) has two MO_6_ octahedra and three PO_4_ tetrahedra sharing oxygen atoms for linkage, with the Na^+^ occupying two unequal Wyckoff sites, one Na^+^ at the 6b site (M1) and the other at the 18e site (M2). The pyrophosphate-type materials are represented as Na_2_MP_2_O_7_ (M = V, Fe, Co), with P_2_O_7_^5–^ having a higher inductive effect than PO_4_^3–^ that can greatly increase working voltage. In the crystal structure of Na_2_FeP_2_O_7_, the Fe_2_O_11_ copolymer connected by two FeO_6_ octahedra coangularly and P_2_O_7_ connected by two PO_4_ tetrahedra coangularly are bridged together in a coedge or coangle to form a 3D twisted zig-zag-type Na^+^ transport channel, and five Na sites with different occupancy degrees are generated. The redox reaction of Fe^2+^/Fe^3+^ occurs at a suitable voltage window for the reversible extraction/insertion corresponding to one Na^+^ with a theoretical specific capacity of 97 mA h g^–1^, and two clear plateaus of 2.5 and 3.0 V can be observed.

#### 3.1.2 Sulfates

The thermodynamic stability of the SO_4_^2–^ group in sulfate polyanionic compounds Na_2_M(SO_4_)_2_·*n*H_2_O (M = Fe, Mn, Co, Ni, etc.) is inferior, and its decomposition temperature is lower than 400 °C. When exposed to temperatures above the decomposition temperature, SO_2_ gas is easily released, resulting in low chemistry purity and toxic substances, so the low-temperature solid-phase method is often used for sulfate synthesis. According to the Pauling electromagnetic principle, the bonding of S–O is stronger than that of P–O, leading to the strong induction of sulfate. Thus, the energy level cleavage resulting from the hybridization of the d orbitals of transition metal ions with O 2p orbitals is intensified, making the redox potential of the material high. Since the charge-to-mass ratio of sulfate is significantly lower than that of phosphate, the theoretical specific capacity of sulfate materials is thus lower. Although in terms of practical applications, sulfate materials can hardly be comparable to commercialized LiCoO_2_, LiFePO_4_, NCM-811, and so on, they can unleash their own unique advantages in the field of low-cost energy storage. In 2014, Yamada et al. successfully prepared Na_2_Fe_2_(SO_4_)_3_, which belongs to monoclinic crystal system with the *P21/c* space group.^80^ It possesses a three-dimensional skeleton structure and Na^+^ diffusion channels along the *c*-axis direction for Na^+^ migration, delivering a theoretical capacity of 120 mA h g^–1^ with a high working platform of 3.8 V. As illustrated in Figure 6b, the FeO_6_ octahedra form an isolated Fe_2_O_10_ dimer by coedging and bridging with the SO_4_ tetrahedra through the vertices, thus resulting in a three-dimensional (3D) skeletal structure with large ion channels along the *c*-axis. More specifically, Na^+^ occupies three different Na sites in the 3D skeleton structure, where the Na2 and Na3 sites have 1D Na^+^ diffusion channels along the *c*-axis and the Na^+^ located at these two sites can easily diffuse along their respective channels.

#### 3.1.3 Silicates

Silicates, represented as Na_2_MSiO_4_ (M = Fe, Mn, Co, Ni), are promising cathode candidates for large-scale energy storage because they possess the cost-effectiveness and resourcefulness of Na, Fe, and Si raw materials on Earth. Take Na_2_FeSiO_4_ as an example to dissect the structure of a silicate-based polyanionic compound. In monoclinic Na_2_FeSiO_4_ materials (space group *Pn*), the FeO_4_ tetrahedron and SiO_4_ tetrahedron are joined alternately to form a solid framework, while the sodium is hexacoordinated and forms a sublattice alone; Na ions are in a relatively disordered state in this structure (Figure 6c). Due to the large ion gap in the structure framework, Na^+^ has greater freedom of motion, leading to a high Na^+^ diffusion coefficient. Thus, high Na^+^ diffusion in Na_2_FeSiO_4_ can still be achieved even without the fast ion transport channels, which also applies to Co-based and Mn-based silicate cathodes.^81^ Na_2_FeSiO_4_ with a high theoretical capacity of ∼278 mA h g^–1^ corresponds to reversible insertion/extraction of two Na^+^ per unit. Although theoretical studies showed the possibility of 2e^–^ reactions in silicate electrode materials, current studies show the occurrence of only the 1e^–^ reaction owing to electrolyte decomposition at potentials required to insert/extract the second Na^+^.^82^

#### 3.1.4 Borates

Reports on borate-based materials are much rarer. Borate polyanionic electrode materials have also received some attention from researchers because of their small molar mass, abundant resources, and environmental friendliness. Boron atoms can be sp^2^- and sp^3^-hybridized to form various groups, such as [BO_3_]^3–^, [BO_4_]^5–^, and [B_2_O_4_]^4–^, that can be condensed or polycondensed to form islands, chains, layers and skeletal groups, leading to a variety of boronate crystal structures. Compared with other polyanionic compounds, borates have higher theoretical capacities but a much lower operating voltages due to the weak induction. The pentaborate polyanionic cathode material of Na_3_MB_5_O_10_ (M stands for V, Fe, Mn, Co, etc.) can be easily fabricated by the solid-phase method. As illustrated in Figure 6d, Na_3_FeB_5_O_10_ belonging to the orthogonal crystal structure (space group *Pbca*) consists of four vertices of FeO_4_ tetrahedra connected to the [B_5_O_10_]^5–^ unit, with the FeO_4_–B_5_O_10_ network aggregated into layers in the *ab*-plane and stacked along the *c*-axis; Na^+^ occuplies the interlamination positions.^83^ Additionally, its theoretical capacity is 78 mA h g^–1^ based on the reversible intercalation of one Na^+^ per formula unit.

#### 3.1.5 Mixed-Polyanion Materials

A series of hybrid polyanionic cathode materials with novel structures, such as a phosphate–pyrophosphate hybrid, a phosphate–carbonate hybrid, and fluorinated phosphate, can be obtained by taking advantage of their mutual compatibility. The phosphate–pyrophosphate hybrid polyanionic cathode material can be expressed as Na_4_M_3_(PO_4_)_2_(P_2_O_7_) (M = Fe, Ni, Mn, Co, etc.). The crystal structure of Na_4_M_3_(PO_4_)_2_(P_2_O_7_) is rhombohedral with a space group of *Pn21a*, according to Figure 6e, and the MO_6_ octahedra and PO_4_ tetrahedra form a double chain by covertex connections, which is further bridged to a laminar structure by P–O–P bonding of pyrophosphate.^84^ The Na_4_Fe_3_(PO_4_)_2_(P_2_O_7_) first reported by Kang’s group exhibited excellent Na storage performance as a cathode for SIBs, with a high reversible specific capacity of 129 mA h g^–1^ with operating voltage of ∼3.2 V, achieving energy density of 412.8 W h kg^–1^.^89^

In addition, a series of crucial fluorinated polyanionic materials with high voltages can be prepared by replacing part of the polyanion with high electronegativity fluorine, thereby developing NaVPO_4_F, Na_3_V_2_(PO_4_)_2_F_3_ (NVPF), Na_3_V_2_(PO_4_)_2_O_2_F (NVPOF), NaFeSO_4_F, and other fluorinated materials. The tetragonal NVPF with a space group of *P*4_2_/*mnm* was composed of [V_2_O_8_F_3_] bioctahedra and [PO_4_] tetrahedra that interconnected by angle sharing, and the occupancy ratio between Na(1) sites and Na(2) sites is 2:1. Owing to the high electronegativity of fluorine, the fluorinated material has a high working plateau of ∼3.9 V with a theoretical capacity of ∼130 mA h g^–1^ (corresponding to a theoretical energy density of ∼500 W h kg^–1^), thus being quite suitable for high-energy SIBs.^85,86^

### 3.2 Promising Polyanionic Cathodes

#### 3.2.1 Na_3_V_2_(PO_4_)_3_

**Figure 7:**
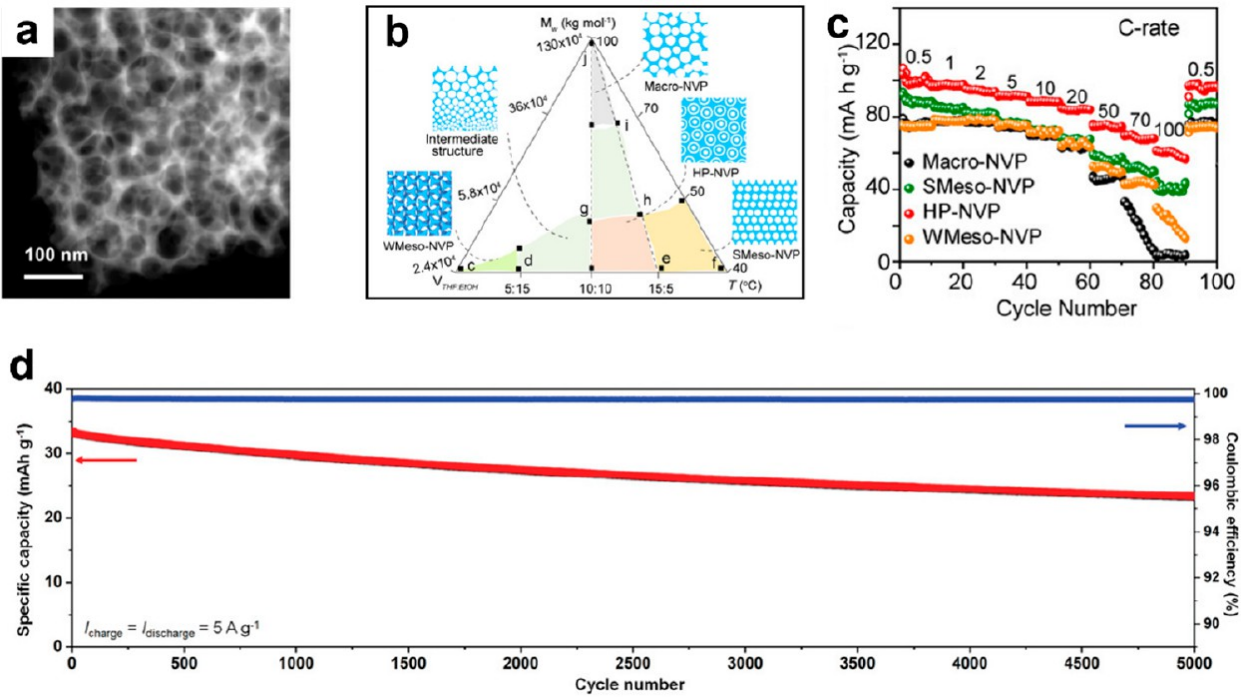
(a) TEM of the hierarchically porous Na_3_V_2_(PO_4_)_3_ materials and (b) ternary diagram of pore structure of porous NVP. (c) Rate capacities of the Na_3_V_2_(PO_4_)_3_ materials with different pore morphologies. Reproduced with permission from ref (91). Copyright 2021 Wiley-VCH. (d) Cycling performance of NVP@C@CNTs||NaPF6 in Diglyme||MCMB at 5 A g^–1^. Reproduced with permission from ref (92). Copyright 2022 Wiley-VCH.

As one of the most widely studied polyanionic materials, Na_3_V_2_(PO_4_)_3_ (NVP) possesses high ionic conductivity, excellent cycling stability and great thermal stability. Employed as the cathode for SIBs, Na_3_V_2_(PO_4_)_3_ has a theoretical capacity of 117.6 mA h g^–1^ and a working plateau of 3.4 V, which originated from the V^3+^/V^4+^ redox couple corresponding to two Na^+^ ionsinvolved in (de)sodiation.^87,90^ Unfortunately, the NVP cathode material characterized by sluggish diffusion kinetics and low electronic conductivity (∼10^–12^ cm^2^ s^–1^) has an unsatisfactory specific capacity and rate capability, and effective modifications are desired. At this stage, modification methods, including surface coating, morphological construction, and lattice modulation, have been developed. Recently, Xiong and coworkers proposed a polymer-stabilized droplet template strategy to synthesize a novel porous single-crystal-structured Na_3_V_2_(PO_4_)_3_ compound (Figure 7a), and selected area electron diffraction (SAED) confirmed its single-crystal structure. The phase diagram in Figure 7b summarizes the pore structures at the mesoscale and macroscopic scales under various reaction conditions. When less volatile solvents combine with high-molecular-weight polyvinylpyrrolidone (PVP), hierarchically meso/macroporous structured NVP could be synthesized. Compared with the macroporous and mesoporous structures, the hierarchically porous structure with the 3D interlinked channel provides faster Na^+^ transport paths and a larger contact area, effectively accelerating the Na^+^ transportation. Another advantage is that Na^+^ can migrate along the smooth solid–liquid interface in the HP-NVP. As a consequence, an outstanding rate capability of 61 mA h g^–1^ at ultrahigh rate of 100 C and a prolonged lifespan of 10000 cycles at 20 C without capacity fading can be achieved (Figure 7c). Specially, HP-NVP was assembled to form a symmetric cell, which exhibits a specific capacity of 47 mA h g^–1^ at 50 C and stable cycling at 10 C for 700 cycles.^91^ Xu et al. proposed a spray drying method for synthesizing NVP/rGO HSs. Owing to the unique porous hollow architecture effectively shortening the Na^+^/e^–^ diffusion path, the synthesized NVP/rGO HSs compound manifested a high reversible capacity of 116 mA h g^–1^ at 1 C (98% of theoretical capacity), an outstanding high-rate capability of 98.5 mA h g^–1^ at 20 C, as well as a stable cycling performance of 73.1 mA h g^–1^ over 1000 cycles at 10 C. To explore the practical application of NVP/rGO Hs cathode, the full cells assembled with NVP-HSs cathode and S-CMTs anode exhibit a capacity retention of 84.2 mA h g^–1^ after 100 cycles at 1 C. Assembling a high-performance sodium-ion full battery (SIFB) requires overall matching between the cathode, anode and electrolyte. Wei et al. proposed an excellent SIFB integrated with an optimized NVP@C@carbon nanotube (NVP@C@CNTs) cathode, a mesocarbon microbead (MCMB) anode, and a Na^+^–diglyme electrolyte. The as-synthesized NVP@C@CNT cathode displays a high electronic conductivity, reducing the overpotential and charge transfer resistance and leading to a superior rate capability at a high rate of 80 A g^–1^. Besides, it demonstrated a discharge capacity of 70 mA h g^–1^ with extraordinary stability over ultralong 20 000 cycles at a high current density of 20 A g^–1^. Furthermore, the NVP@C@CNTs||MCMB full cell obtained high energy density of 88 W h kg^–1^ at ∼10 kW kg^–1^ and 58 W h kg^–1^ at ∼23 kW kg^–1^. Besides, superior cyclability with 72.7% capacity retention for 5000 cycles at 5 A g^–1^ could be achieved (Figure 7d). Both the high conductivity of NVP@C@CNT cathode and the expanded ion diffusion paths at the anode resulted from the initial pseudocapacitive cointercalation, which contributed to this high rate capability and excellent cyclability.^92^

In addition to morphological construction and surface coating, lattice regulation is also beneficial for improving NVP performance. Most studies primarily concentrate on introducing inactive elements into the V site. Liang et al. fabricated Mo-doped 1D NVP nanowires (MNVP@C NWs) as a multifunctional cathode for Na storage and fully explored their practicality.^93^ The electron energy loss spectroscopy (EELS) confirmed the uniform distribution of external Mo within the nanowires rather than surface doping. This cathode obtained a discharge capacity of 116.8 mAh g^–1^ at 0.1 C and displayed a capacity retention of 85.7% after 8000 cycles at 5 C. Additionally, the constructed pocket-flexible SIBs demonstrated a large energy density of 262.4 W h kg^–1^ and an ultrahigh rate capability of 77 mA h g^–1^ at 150 C. This is because when higher valence Mo^6+^ was introduced into the NVP, Na^+^ vacancies would be generated due to valence equilibrium, which enhanced the electronic conductivity and ion diffusion kinetics of the electrode due to the smaller Na^+^ migration barrier. Besides, Shi et al. developed a cathode of bismuth-doped NVP enwrapped with carbon nanotubes.^94^ The optimized Na_3_V_1.97_Bi_0.03_(PO_4_)_3_/C@CNTs sample displayed a reversible capacity of 97.8 mA h g^–1^ and maintained a capacity of 80.6 mA h g^–1^ over a prolonged 9000 cycles at 12 C. Even when cycled at an ultrahigh rate of 80 C, the cathode also exhibited a high capacity of 84.3 mA h g^–1^ and achieved 87% of its capacity after 6000 cycles. These excellent rate capability and outstanding cyclability can be attributed to the doped Bi^3+^ that acted as the pillar of NVP crystal structure, buffering crystal deformation and enhancing the structural stability.

#### 3.2.2 Na_3_VM(PO_4_)_3_

**Figure 8:**
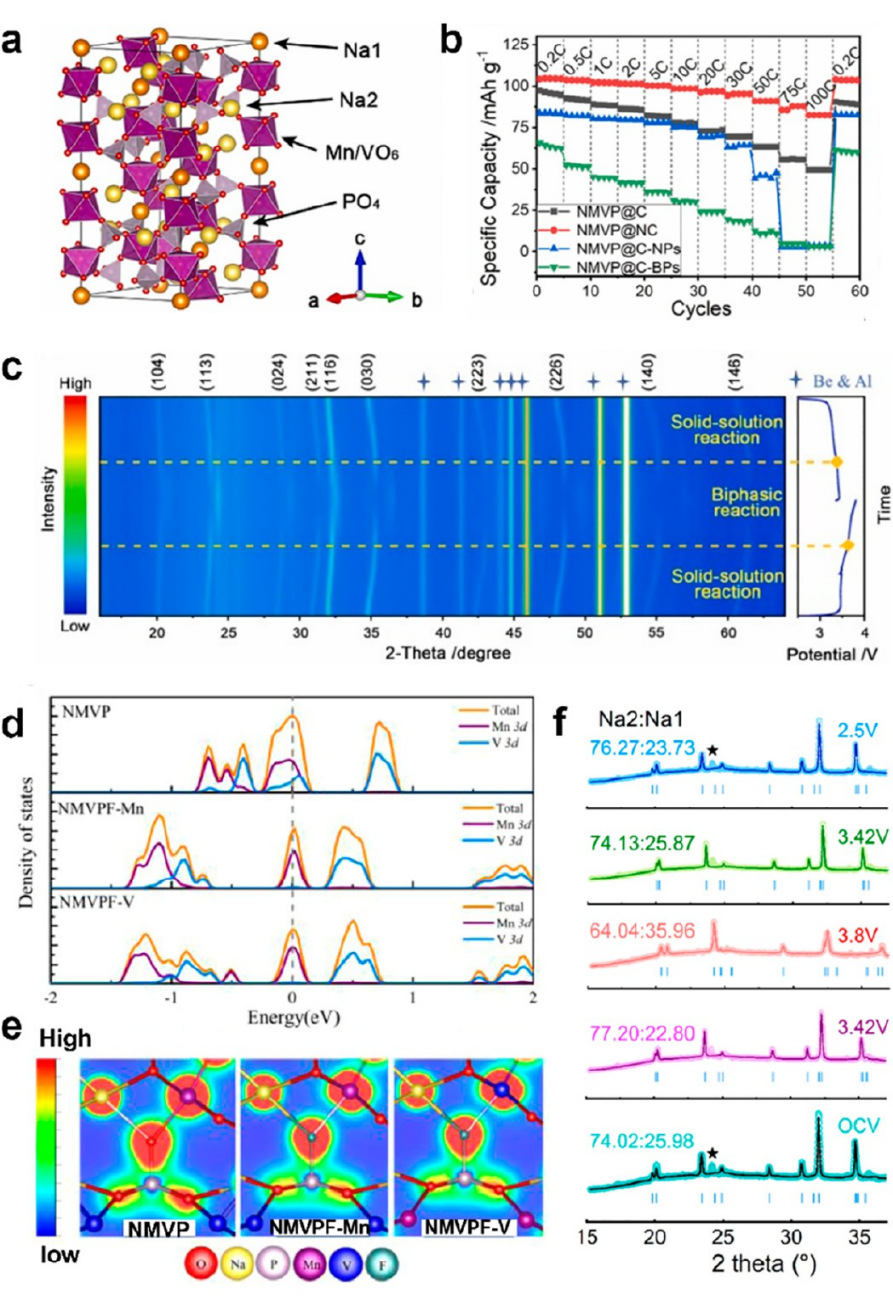
(a) Crystal structure of Na_4_MnV(PO_4_)_3_, (b) rate performance of the NMVP-based cathode, and (c) in situ XRD pattern of Na_4_MnV(PO_4_)_3_@NC. Reproduced with permission from ref (96). Copyright 2022 Elsevier. (d) Total and projected density of states and (e) side-view of the electron density difference of NMVP and NMVPF-Mn/V samples. (f) Rietveld refinement of partial in situ XRD patterns of NMVPF. Reproduced with permission from ref (98). Copyright 2021 Elsevier.

Vanadium-based materials profiting from multivalence states and rich resource of vanadium are some of the preferred electrodes for batteries, but vanadium has high toxicity. Thus, cost-efficient and environment-friendly elements (e.g., Fe, Mn) are doped into the V-site in Na_3_V_2_(PO_4_)_3_, producing Na_4_VFe(PO_4_)_3_ and Na_4_VMn(PO_4_)_3_. As illustrated in Figure 8a, Na_4_VMn(PO_4_)_3_ is constructed by MnO_6_/VO_6_ octahedra sharing all the corners with PO_4_ tetrahedra, and it possesses a theoretical capacity of 111 mA h g^–1^ and voltage plateaus of 3.6 (Mn^2+^/Mn^3+^) and 3.3 V (V^3+^/V^4+^) corresponding to reversible insertion/extraction of two Na^+^. Except for suffering from low electron migration kinetics, the John–Teller effect of Mn^3+^ will lead to Mn digestion and structural instability, shortening the lifespan of the Na_3_VM(PO_4_)_3_ electrode.^95^ The most facile and effective way to improve the electronic conductivity of Na_3_VM(PO_4_)_3_ is to coat it with conductive materials. Recently, Zhu et al. designed a unique hierarchical bayberry-like NMVP@NC material as a cathode for SIBs via facile ball-milling and subsequent calcination. Even cycled at an ultrahigh rate of 100 C, the NMVP@NC cathode can still deliver a high discharge capacity of 82.4 mA h g^–1^ (Figure 8b), which is far superior to other NMVP-based electrodes. When assembled with commercial soft carbon as the anode, the full cell could deliver 94 mA h g^–1^ at 0.1 C and 57 mA h g^–1^ at 10 C. Figure 8c reveals the structural evolution of the NMVP@NC cathode during the first electrochemical cycle. The NMVP@NC cathode underwent a solid-solution reaction when charged to 3.6 V and a biphasic reaction in the interval of 3.6–3.8 V. Besides, peaks during the discharge and the charge process appear symmetric, confirming the high reversibility of the electrochemical reactions. The NMVP@NC cathode is unique: (i) the ultrasmall sizes of nanoparticles render a short diffusion distance for Na^+^ and provide a larger electrode/electrolyte contact area, (ii) the 3D N-doped carbon network availably improves the electrical conductivity of NMVP, and (iii) the robust structure suppresses the volumetric expansion during the repeated Na^+^ insertion/extraction, giving rise to superior cyclability.^96^

To mitigate the malignant effects caused by Mn^3+^, more modification of Na_4_MnV(PO_4_)_3_ associated with heteroatomic doping of Al^3+^, Mg^2+^, and other elements has been explored. The substitution by heteroatoms is aimed at reducing the concentration of Mn^3+^ in the NVMP cathode so that the Jahn–Teller distortion is suppressed and structural stability is enhanced with increased covalency by inducing shorter (V/Mn/Mg/Al)–O bond lengths. Moreover, the substitution of inert Al^3+^ into the NVMP structure would generate abundant Na vacancies, which are expected to reduce the activation energy and enhance the Na^+^ mobility.^97^ In addition to doping at the vanadium sites, doping at the polyanion sites has also been studied. The innovative Na-deficient Na_3.85_□_0.15_MnV(PO_3.95_F_0.05_)_3_ material was fabricated by partially doping F into the NMVP. Electron density differences shown in Figure 8d and e prove that the change of electron density caused by the substituted F near Mn or V atoms and the weak Coulomb interaction induced by the Na vacancy effectively promotes the Na^+^ diffusion dynamics. Executing ex situ ^23^Na NMR and in situ XRD (Figure 8f) characterization of NMVPF electrodes at different states of (dis)charge revealed the higher Na^+^ extraction rate from the Na2 site.^98^

**Figure 9:**
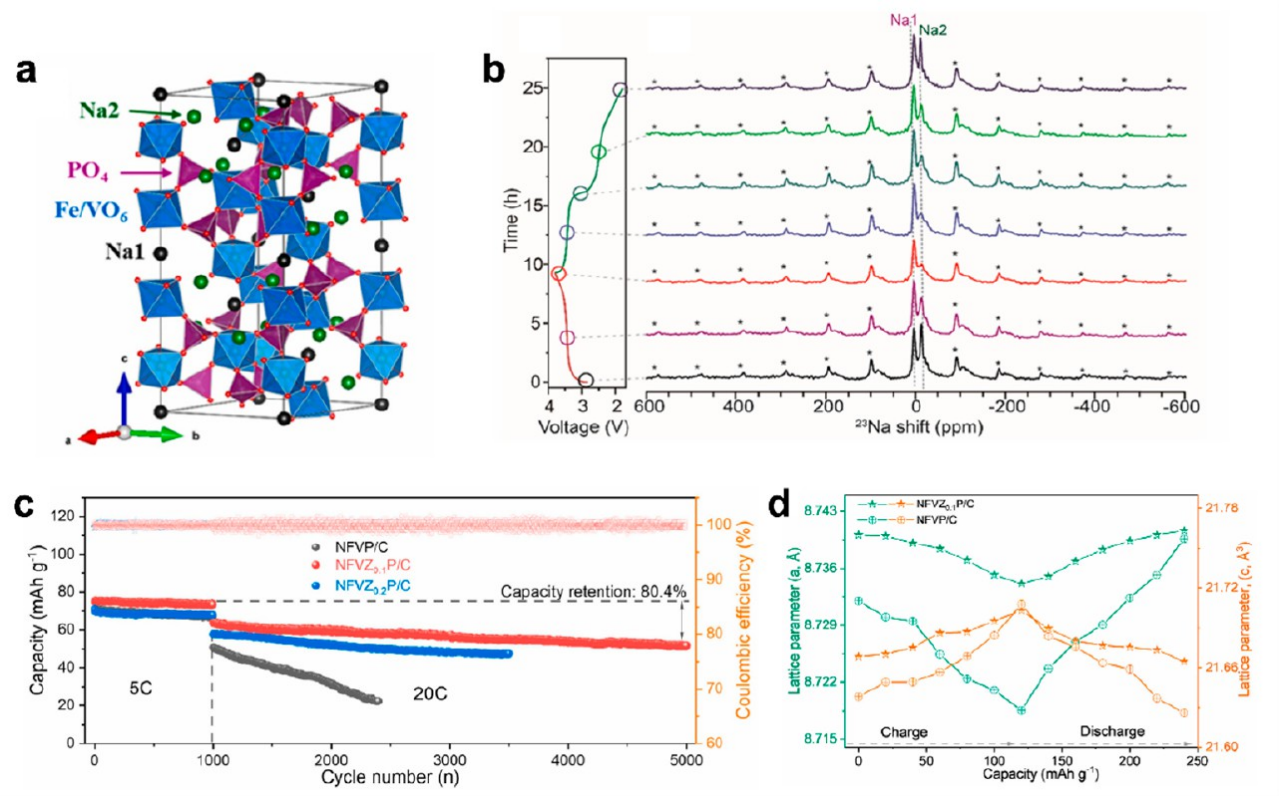
(a) Crystal structure of Na_4_FeV(PO_4_)_3_. (b) ^23^Na magic-angle-spinning (MAS) NMR of NFVP@C at different cycle states. Reproduced with permission from ref (99). Copyright 2022 Elsevier. (c) Cycling performance of NFVP/C with varying Zr-content (0–0.2). (d) Variation of the lattice parameters of NFVP/C and NFVZ_0.1_P/C electrodes. Reproduced with permission from ref (101). Copyright 2022 Elsevier.

Na_4_FeV(PO_4_)_3_ belonging to the Na_4_VM(PO_4_)_3_ type is another promising cathode for SIBs with a similar framework as Na_4_MnV(PO_4_)_3_. It is constructed by FeO_6_/VO_6_ octahedra with PO_4_ tetrahedra (Figure 9a). Lu et al. proposed a novel Na_4_FeV(PO_4_)_3_@C cathode synthesized via a combined ball-milling, sol–gel, and calcination process. The as-prepared Na_4_FeV(PO_4_)_3_@C exhibited specific capacities of 100 mA h g^–1^ at 0.1 C and 80.6 mA h g^–1^ at 10 C when tested at the wide voltage window of 1.3–3.8 V. In charge–discharge curves, two plateaus located at 2.5 and 3.5 V can be ascribed to Fe^2+^/Fe^3+^ and V^3+^/V^4+^ redox couples, respectively. Besides, the cathode exhibited great cycling stability, with 96.8% capacity retention after 800 cycles at 5 C, surpassing the original Na_4_FeV(PO_4_)_3_ cathode that rapidly decays after only 400 cycles. The solid-state ^23^Na nuclear magnetic resonance revealed that the Na^+^ stand at Na2 sites exhibited faster insertion/extraction dynamics upon cycling (Figure 9b). XRD and time-of-flight neutron powder diffraction illustrated that the electrochemical process undergoes a reversible solid-solution reaction, confirming its stable framework structure.^99^ Wang et al. reported a bicarbon-decorated NFVP@rGO@CNT material as the cathode for SIBs. A high discharge capacity of 156 mA h g^–1^ at 0.1 C could be achieved in the operating window of 2.0–4.4 V. Besides, a rate capacity of 60 mA h g^–1^ at 30 C and 71% capacity retention over 600 cycles at 2 C were realized. Such great rate performance and cyclability benefit from the double carbon layer (CNTs and rGO) accelerating the electron transfer. Even when fabricated with a high mass loading of 6.2 mg cm^–2^, the cathode exhibited excellent rate capability and cyclability (58.7 mA h g^–1^ at 30 C and 72.1% capacity retention after 1000 cycles at 10 C), confirming its practical potential.^100^ Ma et al. reported a heteroatomic doping strategy in Na_4_FeV(PO_4_)_3_ materials, which generate extra Na vacancies to boost the electroconductivity. The optimized Na_3.9_FeV_0.9_Zr_0.1_(PO_4_)_3_/C electrode exhibited a high discharge capacity (114 mA h g^–1^ at 0.1 C), superior rate capability (66.7 mA h g^–1^ at 40 C), and remarkable cyclability of 82.4% capacity retention over 4000 cycles at 20 C (Figure 9c). As illustrated in Figure 9d, the NFVZ_0.1_P/C cathode showed a smaller volume change (Δ*V*/*V*_pristine_) of ∼5.21% during electrochemical cycling compared with the undoped sample. Its excellent structural stability benefited from the pillar support from Zr. Furthermore, the assembled NFVZr_0.1_P/C||HC full cell exhibited a superior rate capacity of 56.4 mA h g^–1^ at 1000 mA g^–1^ and 97% capacity retention over 100 cycles at 200 mA g^–1^.^101^

More recently, a high-entropy crystal substitution strategy for promoting polyanionic materials was proposed by Li and coworker, and Na_3_VAl_0.2_Cr_0.2_Fe_0.2_In_0.2_Ga_0.2_(PO_4_)_3_ (denoted as NVMP) was developed via a facile sol–gel method and explored for SIBs at both ambient and low temperatures. Benefiting from the doping of high-entropy crystals, the activity of V^4+^/V^5+^ electron couples is activated, enabling a highly reversible capacity of 102 mA h g^–1^ at 0.1 C. Besides, the NMVP half-cell also showed outstanding cyclability over 5000 cycles at 20 C. Even tested at −20 °C, the NVMP cathode could still demonstrate prolonged cyclability with 94.2% capacity retention over 1000 cycles at a high rate of 5 C. For a real application, the constructed NVMP||HC full batteries could deliver 81 mA h g^–1^ at 0.2 C and steadily cycle for 50 cycles when paired with hard carbon.^102^

The element doping strategy for polyanionic compounds, particularly vanadium-based phosphates, focuses on using more cost-effective transition metal elements (e.g., Mn, Fe, etc.) to replace V, achieving a lower vanadium content while maintaining similar electrochemical performance. Mn substitution brings about higher voltage plateaus (3.6 V), but it also generates an unfavorable Jahn–Teller effect. To address this, additional atoms such as nonactive metal elements like Al, Mg, Ce, and Cr are introduced to suppress the adverse effects caused by Mn^3+^.^103^ The introduction of Fe stabilizes the lattice further, but the overall decreased voltage (∼3.0 V) limits its application in high-energy-density SIBs, making it less favorable. On the other hand, the introduction of highly electronegative fluorine at polyanion sites has proven to be an effective strategy for promoting the working voltage of polyanionic compounds. However, it is crucial to note that excessive F content hinders the transmission of Na^+^ in the lattice, necessitating strict control over its introduction amount. Furthermore, utilizing high-entropy crystal substitute and activating electron redox with higher valence states are promising avenues for future research.

### 3.3 Scalable Preparation of Polyanionic Compounds

The scalable preparation of polyanionic materials is also of great concern. Qi et al. proposed a groundbreaking synthesis route for the scalable production (150 g per batch) of multishelled Na_3_(VOPO_4_)_2_F microspheres using in situ generated bubbles as soft templates at room temperature.^104^ In this method, raw materials were extracted from vanadium slag and NVPOF microspheres were formed during the coprecipitation process with the appropriate reaction time. The large-scale prepared Na_3_(VOPO_4_)_2_F exhibited an outstanding rate capacity of 81 mA h g^–1^ and remarkable cycling stability, with 70% capacity remaining over 3000 cycles at 15 C. This room-temperature scalable production strategy paves the way for the commercialization of SIBs. Similarly, Shen and coworker developed a rapid synthesis route for Na_3_V_2_(PO_4_)_2_O_2_F using a solvent-free room-temperature solid-phase mechanochemical method, and they rigorously verified the feasibility of production using ten different types of vanadium raw materials.^105^ The optimized NVPOF@8%KB demonstrated a high initial capacity (142.2 mA h g^–1^ at 0.1 C), superior rate capability (112.8 mA h g^–1^ at 20 C), and remarkable cyclability (maintaining 98% capacity over 10 000 cycles at 20 C). To confirm the feasibility of large-scale production of sodium vanadium fluorophosphate using mechanochemical methods, a kilogram-scale preparation was executed. Subsequently, the large-scale synthesized NVPOF materials were matched with a hard carbon anode to fabricate a 26650 cylindrical battery, which delivered a high capacity of 1500 mA h g^–1^ and an energy density of ∼90 Wh kg^–1^. The successful kilogram-scale production and the excellent electrochemical performance of the large-scale synthesized product further validate the feasibility of the mechanochemical method for the commercial SIB cathode materials.

## 4 Prussian Blue Cathodes

Hexacyanoferrates (HCFs)/Prussian blue (PB) and its analogues (PBAs) are promising cathode candidates for SIBs owing to their low cost, easy preparation, and open framework structure for Na^+^ accommodation.^6,106,107^ The chemical formulas of PBAs could be denoted as Na_*x*_M_1_[M_2_(CN)_6_]_*y*_□_1–*y*_·*z*H_2_O (0 ≤ *x* ≤ 2, 0 ≤ *y* ≤ 1), where M_1_ = Fe, Mn, Ni, Cu, Co, Zn, etc.; M_2_ = Fe, Mn, Co; and □ represents a transition metal coordinated with N and C atoms and [M_2_(CN)_6_] vacancies inside the crystal structure, respectively.^108^ The crystal structures of PBAs can be cubic, monoclinic, rhombohedral, and trigonal, which vary according to the number of Na^+^ ions and the content of water. Generally, the alkaline-deficient PBAs present a cubic structure, while alkaline-rich PBAs show a monoclinic phase.^109^ After dehydration treatment, the phase structure will be transformed to rhombohedral or trigonal for the reduced amount of water.^110^ The specific capacities of PBAs depend on chemical compositions when applied as cathode materials for SIBs (e.g., 85 mA h g^–1^ for a single-electron redox-active site (SE-PBAs M_1_ = Zn, Ni) and 170 mA h g^–1^ for double-electron redox-active sites (DE-PBAs, M_1_ = Mn, Fe, Co)). Taking into account the high average discharging voltage (above 3.0 V vs Na^+^/Na), the theoretical energy density of DE-PBAs could reach 510 Wh kg^–1^, which is competitive with commercial LiFePO_4_ employed in LIBs.^111^

Typically, PBAs are prepared by simple coprecipitation of sodium hexacyanoferrate and transition metal salts in water. However, the obtained PBAs present a random distribution of H_2_O and [M_2_(CN)_6_] vacancies because of the rapid reaction between the hexacyanoferrate ligand and transition metal ions.^12^ Additionally, the H_2_O in PBAs can be divided into three species: (i) H_2_O adsorbed on the surface, (ii) interstitial or zeolite H_2_O located at the alkali metal ion sites, and (iii) coordinated H_2_O chemically bonded with transition metals for the absence of [M_2_(CN)_6_]. The H_2_O/vacancies in PBAs would cause lattice distortion and even structure collapse during (de)sodiation processes, leading to rapid capacity degradation.^112^ Meanwhile, the irreversible phase transition during charging and discharging process also contributes to the short cycle lifespan. Further, low electronic conductivity for poor rate performance of PBAs is another obstacle should be overcome for practical application.^113^ As a consequence, strategies aiming at preparing PBAs with low levels of water and vacancies, mitigated phase transitions during cycling, and enhanced electronic conductivity are imperative and challenging.

### 4.1 Modification of Prussian Blue Materials

Although many metals are capable of occupying the M_1_ and M_2_ sites, the Fe-based (M_1_ = M_2_ = Fe) and Mn-based (M_1_ = Mn, M_2_ = Fe) PBAs with two redox-active centers are the most investigated for their high theoretical specific capacity and low cost. Besides of the high content of H_2_O/[Fe(CN)_6_] vacancies and low electronic conductivity, Fe-based PBAs also suffer from a low practical specific capacity for the irreversible electrochemical reaction of low-spin Fe coordinated with C,^114^ and the Mn-based PBAs suffer from the Jahn–Teller effect of Mn^3+^ and the dissolution of Mn^2+^.^115,116^ Crystal structure control, nonaqueous preparation/dehydration treatment, compositing with conductive carbon, surface coating, and cationic doping are effective approaches to prepare PBAs with low H_2_O/vacancy contents, high crystallinity, high electronic conductivity, and improved the electrochemical performance according to the previous studies, which are summarized in this section. In addition, the scalable preparation of PBAs and its practical application in full-cells were also introduced.

#### 4.1.1 Crystal Structure Control

[Fe(CN)_6_] vacancies inside the PBA crystal were generated during the fast coprecipitation between metal salts and sodium hexacyanoferrate. The vacancies occupied by coordinated water and interstitial water reduce the amount of extractable sodium, hinder the migration of Na^+^, and decrease the practical specific capacity.^117,118^ Additionally, the crystal structure of defect-rich PBAs tends to collapse during (de)insertion of Na^+^ due to the absence of bulky [Fe(CN)_6_], which deteriorates the electrochemical performance.^15^ It is documented that the critical point for decreasing the [Fe(CN)_6_] vacancies and enhancing the crystallization of PBAs is slowing down the reaction rate of coprecipitation. Pioneer researchers have put forward several effective strategies to reduce the rate of coprecipitation: (i) Implementing chelating agent/surfactant-assisted precipitation. A chelating agent, such as sodium citrates (Na_3_Cit),^119^ ethylenediaminetetraacetic acid disodium (Na_2_EDTA),^120^ diethylenetriaminepentaacetic acid disodium (Na_2_DTPA),^121^ and pyrophosphoric salts (Na_4_P_2_O_7_)^122,123^ having high complexation with transition metal salts could slow down the release rate of metal ions and the crystallization ratio. (ii) Lowering the precipitation temperature for Fe-based PBAs. Our group^124^ found that Fe-based PBAs prepared below 0 °C or iced conditions exhibited fewer [Fe(CN)_6_] vacancies than those synthesized at room or high temperature for the decreased reaction rate, which was consistent with the result of Ma’s group.^125^ (iii) Preparing a high salt concentration. Guo’s group reported Mn-PBAs fabricated using a saturated Na_4_Fe(CN)_6_ solution displayed only 4% vacancies (24% at traditional condition), and vacancy-free Mn-PBAs could be obtained once the sample was aged at 80 °C for 20 h.^126^

#### 4.1.2 Nonaqueous Preparation/Dehydration Treatment

Although several reports demonstrated that the interstitial water located at body-centered sites can stably exist in PBAs for structural stabilization,^127^ most researchers believed that the deteriorated electrochemical properties of PBAs are due to the side reactions between water and the nonaqueous electrolyte.^128^ It was found in our group that the water content of the PBA cathode at the discharging state could reach the ultrahigh value about 20 ppm, which was fivefold higher than those in Na_3_V_2_(PO_4_)_3_, and resulted in the swell of pouch cell.^112^ Therefore, it is imperative to solve the water problem of PBAs to promote their commercial application. Normally, preparing PBAs in a nonaqueous solution and performing a dehydration treatment after primary drying are effective methods to reduce the water content.

##### 4.1.2.1 Nonaqueous Preparation

As we discussed above, PBAs were prepared in an aqueous solution, making it difficult to completely eliminate the crystal water (10–15 wt %). Substitution of partial/whole water by organic solvents has been proven effective to suppress crystal water growth in PBAs. You’s group demonstrated that high-crystallinity PBAs with a low water content (7.90%) could be synthesized by the solvothermal method using an ethylene glycol/water mixed solvent to minimize water content in the reaction environment and decrease the crystal nucleation rate.^129^ However, water in the reaction was necessary to dissolve Na_4_Fe(CN)_6_ precursors. To improve the solubility of the precursor and accelerate the reactions in organic solutions, He’s group developed a microwave-assisted solvothermal approach with anhydrous ethanol as the solvent. The microwave supplies external energy, and PBAs could be synthesized at a slightly elevated temperature within hours.^130^ As a result, the content of interstitial water in obtained samples is only 4.34–5.13 wt %, and a high discharging specific capacity of 150 mA h g^–1^ could be reached, suggesting organic solvents are alternative mediums for the preparation of PBAs. Recently, our group proposed a “water-in-salt” nanoreactor strategy to prepare highly crystallized Mn-based PBAs with a decreased water content (10.1% vs 18.5% prepared by coprecipitation), higher volume yield, and enhanced electrochemical performance over a wide temperature range from −10 to 50 °C, indicating it was a promising route to achieve the large-scale production of PBAs.^131^

##### 4.1.2.2 Dehydration Treatment

**Figure 10:**
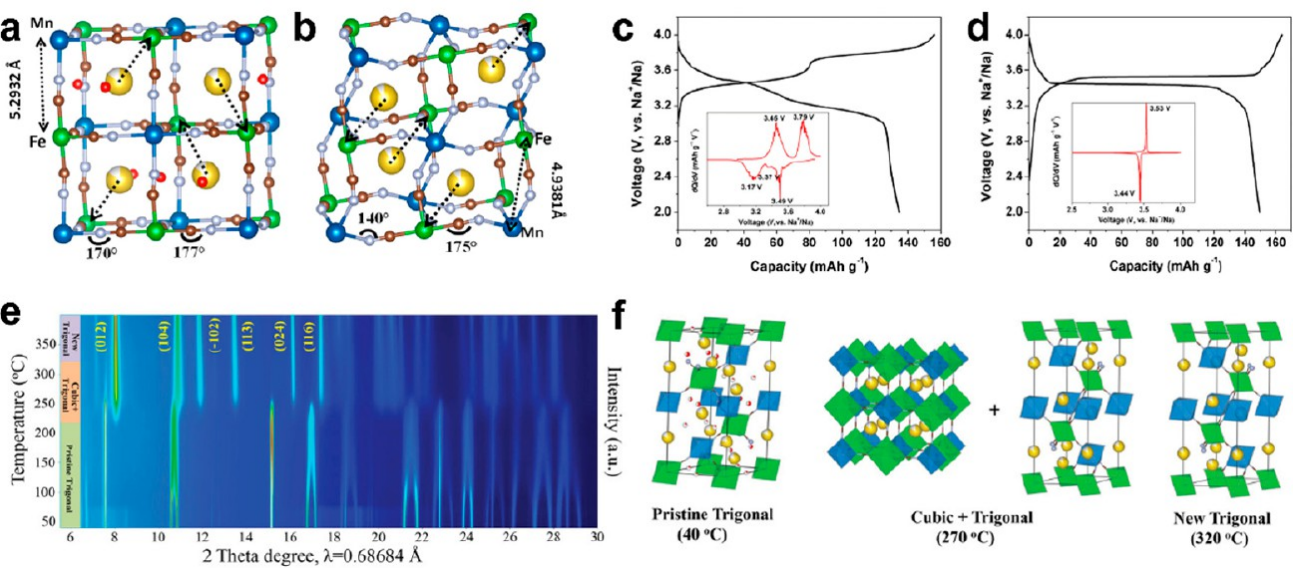
Local structures of (a) M-Na_2−δ_MnHCF and (b) R-Na_2−δ_MnHCF and galvanostatic initial charge and discharge profiles of (c) M-Na_2−δ_MnHCF and (d) R-Na_2−δ_MnHCF. Reproduced with permission from ref (110). Copyright 2015 ACS publications. (e) 2D contour map of in situ high-temperature synchrotron X-ray diffraction patterns of as-prepared Na_2–x_FeFe(CN)_6_ samples under Ar and (f) corresponding crystal structures at 40, 270, and 320 °C. Reproduced with permission from ref (112). Copyright 2022 Wiley-VCH.

Goodenough’s group first proposed the post-dehydration of Mn-based PBAs at low temperature (100 °C) heating under high-vacuum (15 mTorr) for the removal of interstitial water.^110^ After the dehydration treatment, the water content in Na_2−δ_MnHCF decreased from 12% to 2%, indicating the *z* value was only 0.3 in dehydrated Na_2−δ_MnHCF. Meanwhile, the monoclinic Na_2−δ_MnHCF (M-Na_2−δ_MnHCF) converted to the rhombohedral phase (R-Na_2−δ_MnHCF) after the dehydration treatment due to lattice shrinking and distortion, as shown in Figure 10a and b. The electrochemical behavior was also changed after the removal of interstitial water. The M-Na_2−δ_MnHCF electrode show two pairs redox peaks located at 3.17/3.45 and 3.49/3.79 V, and a discharging capacity of 137 mA h g^–1^ could be delivered (Figure 10c), while R-Na_2−δ_MnHCF displayed an apparently single flat plateau at 3.44/3.53 V and a higher initial discharging capacity of 150 mA h g^–1^ (Figure 10d). Additionally, the dehydrated Na_2−δ_MnHCF exhibited promising cycling performance (75% capacity retention after 500 cycles) and rate capability (81% capacity retention at 20 C). A similar conclusion has been drawn by Younesi’s group, that is, the phase structure of Fe-PBAs converted from monoclinic to rhombohedral after dehydration.^132^ Furthermore, the relationship between the water content and phase structure of sodium-rich Na_2–*x*_FeFe(CN)_6_ was systematically studied in our group.^112^ As depicted in Figure 10e and f, the pristine trigonal phase was maintained when adsorbed water was removed (<150 °C), while cubic and new high-temperature trigonal phases could be found from 220 to 300 °C; the trigonal phase dominated at 270 °C, at which the interstitial and coordinated water faded away. The cubic phase disappeared at temperatures higher than 300 °C, and the new trigonal phase was stable up to 400 °C. After dehydration at 270 °C under Ar, the low-spin Fe^2+^/Fe^3+^ redox reaction at ≈3.4 V was activated and the specific capacities were improved. Moreover, the dehydrated Na_2–*x*_FeFe(CN)_6_ exhibited excellent cycling performance (98.9% capacity retention after 2000 cycles at 100 mA g^–1^), evidencing that post-dehydration was an effective strategy to reduce the water content and improve the electrochemical performance of PBAs.

#### 4.1.3 Compositing with Conductive Carbon

Although the 3D open framework inside the PBAs facilitated Na^+^ diffusion, the rate capability of PBAs is below expectation for their limited electronic conductivity. Compositing PBAs with conductive carbon has been considered as an effective strategy to improve their rate performance. Hence, considerable research has been devoted to building mesoscopic or nanoscopic interactions between conductive carbon (carbon nanotubes (CNT),^113^ Ketjen black (KB),^133^ graphene,^134^ ordered mesoporous carbon (CMK-3),^135^ and 3D N-doped ultrathin carbon (3DNC),^136^ among others, and PBAs particles by in situ growth or chemical coprecipitation. Goodenough’s group constructed monodispersed PBAs nanocubes nucleating on a CNT conductive network (PB/CNT).^113^ The “built-in” CNT network accelerated the electron transport and thus the sodiation reaction of PBAs. As a result, PB/CNT delivered a high discharge capacity of 142 mA h g^–1^ at 0.1 C under subzero temperatures (−25 °C), corresponding to 85% capacity retention compared with that at 25 °C. A reversible capacity of 52 mA h g^–1^ at 6 C could be obtained at −25 °C, while it is only 2 mA h g^–1^ for bare PBAs. After that, Dou and coworkers synthesized a PB@C composition through a facile and in situ solution method, with NaFeHCF directly grown on KB chains.^133^ Despite the degraded electrochemical activity of Fe^LS^(C) caused by [Fe(CN)_6_] vacancies, the perfectly shaped PB@C composition with a lower vacancy content (7% vs 15% for bare PB) and fast charge/Na^+^ diffusion exhibited a higher reversible capacity (130 mA h g^–1^ vs 90 mA h g^–1^ at 0.5 C, 1C = 100 mA g^–1^) and unprecedented rate capacity (77.5 mA h g^–1^ at 90 C). Owing to the large specific surface and superior electrical conductivity, 3DNC networks were considered as an ideal skeleton for loading redox-active materials. Zhao’s group found that the Na^+^ adsorption energy at interfaces was decreased and Fe 3d charges were more delocalized after the introduction of 3DNC (8.26 wt %) into NaK-MnHCF, contributing to the better rate performance of the NaK-MnHCF@3DNC composite compared to the bare NaK-MnHCF. Therefore, compositing affords a simple solution to resolve the low electrical conductivity for PBAs.^136^

#### 4.1.4 Surface Coating

**Figure 11:**
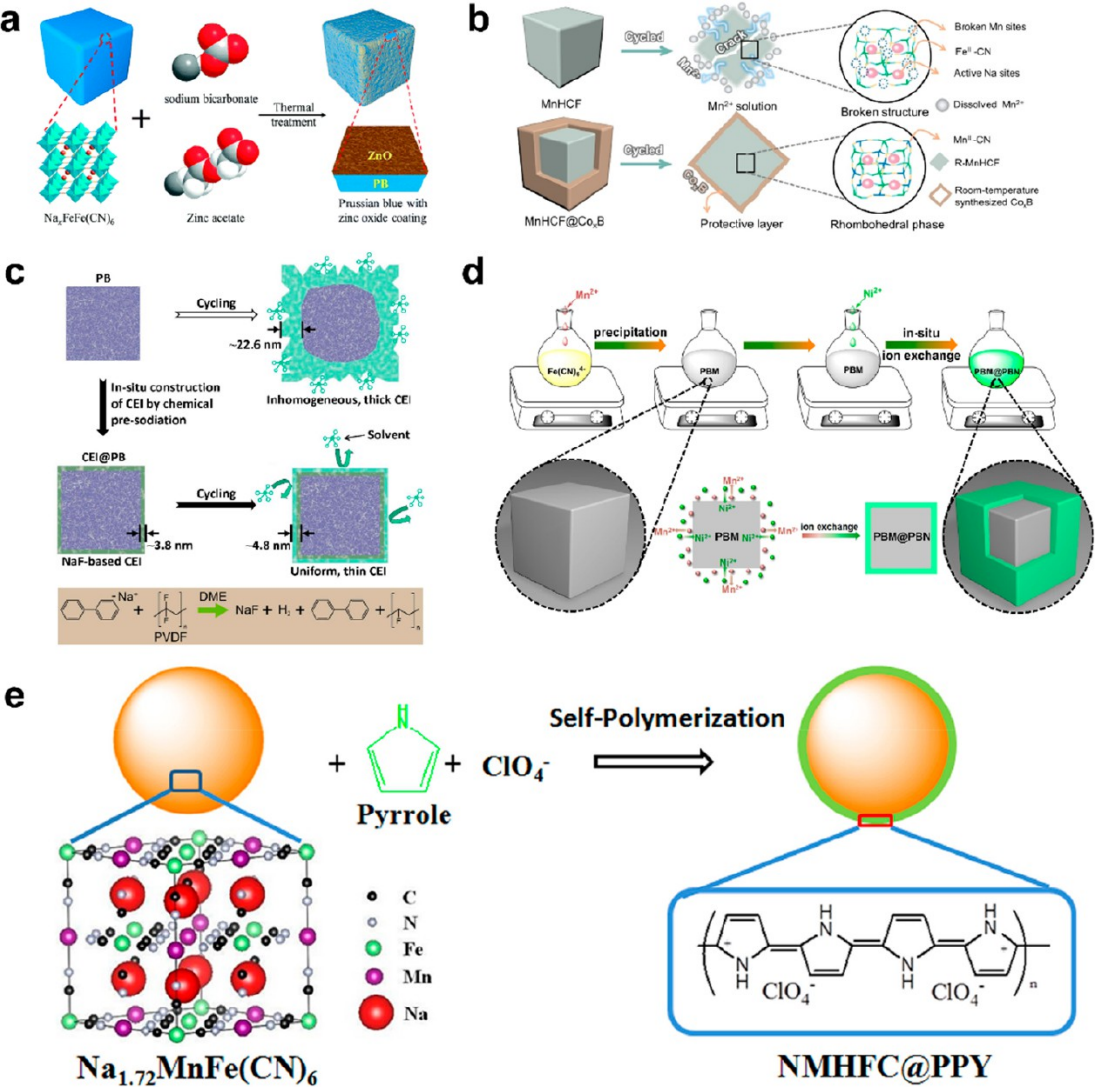
Coating layers for PBAs: (a) ZnO (reproduced from ref (137), copyright 2019 Royal Society of Chemistry), (b) Co_*x*_B (reproduced from ref (138), copyright 2023 Wiley-VCH), (c) artificial NaF-rich CEI (reproduced from ref (139), copyright 2022 Elsevier), (d) Ni-HCF (reproduced from ref (115), copyright 2021 Elsevier), and (e) PPY (reproduced from ref (140), copyright 2015 Elsevier).

It is well acknowledged that PBA cathodes suffer from serious capacity fading due to the transition metal dissolution and side reactions between the electrode materials and organic electrolytes. Thus, surface coating has been used to protect the PBAs from metal dissolution and unwanted side reactions. Considering the instability of PBAs at temperatures higher than 350 °C, however, only low-temperature surface coating is suitable for PBAs. Up to now, inorganic materials, stable SE-PBAs materials (Ni-HCF), and conductive carbon/polymers have been applied as protective layers for electrochemical performance enhancement, as illustrated in Figure 11.

##### 4.1.4.1 Inorganic Materials

Liu’s group constructed a semiconducting and chemically stable ZnO layer (∼50 nm) on the surface of Na_*x*_FeFe(CN)_6_ (PB@ZnO) via a thermal treatment at 200 °C under N_2_ (Figure 11a), which helped reduce the charge-transfer resistance and prohibit the decomposition of the PB lattice.^137^ In order to suppress the microstructural degradation and undesirable Jahn–Teller effect, Hu’s group recently created a magical Co_*x*_B on the MnHCF surface through a facile wet-chemical coating method (Figure 11b).^138^ Owing to the whole coverage of Co_*x*_B, the optimal MnHCF-5%Co_*x*_B cathode displayed limited Mn dissolution and reduced intergranular cracks, thereby contributing to the outstanding cycling performance (74% capacity retention over 2500 cycles at 10 C, 1 C= 170 mA g^–1^). In addition, Ma’s group reported the creation of a Na_3_(VOP_4_)_2_F (NVOPF) coated NaMnHCF composite (PBM@NVOPF) through solution precipitation.^141^ The NASICON-type NVOPF with high chemical stability can undoubtably protect NaMnHCF from the corrosion of HF formed in the electrolyte and inhibit the dissolution of active materials. Hence, excellent electrochemical performance at both room temperature (84.3% capacity retention after 500 cycles) and 55 °C (78.8% capacity retention after 200 cycles) could be acquired at the current density of 100 mA g^–1^.

Moreover, the degradation of PBAs would induce cracks and even the collapse of the cathode–electrolyte interface (CEI), and the newly exposed surface could trigger new CEI formation. Eventually, a thick and uneven CEI was formed and the electrolyte was used up, leading to the death of SIBs. Therefore, it is of great significance to construct a stable and homogeneous CEI on the surface of PBAs to enhance the cycling stability of SIBs. Lately, Li’s group created an artificial NaF-rich CEI via chemical presodiation between metallic Na, biphenyl, and 1,2-dimethoxyethane (DME), as illustrated in Figure 11c.^139^ The Na^+^-conducting NaF-rich CEI effectively prevents CEI@PB from attacking organic solvents and contributes to the longer lifespan of coated PBAs compared to those of bare PBAs. More importantly, the uniformity was maintained, and the thickness of the CEI was approximately 4.8 nm after cycling, which was much smaller than that of PB (∼22.6 nm).

##### 4.1.4.2 Stable SE-PBAs Materials

Although the aforementioned inorganic coating layers have been proven effective, a lattice mismatch between PBAs and coating exists. Coating PBAs with compounds of similar lattice parameters will eliminate lattice mismatches to a greater extent. Among various PBAs with different transition metal ions, Ni-based PBAs (Ni-HCF) have been proven the most chemical/electrochemical stable materials with “zero strain” during cycling.^142^ Therefore, most research focused on preparing a core–shell composite with Ni-HCF as the outer shell to suppress lattice distortion, transition metal dissolution, and the side reactions between PBAs and the organic electrolyte, thus enhancing the electrochemical performance. Generally, the following three strategies have been adopted for Ni-HCF coating: (i) In situ deposition of Ni-HCF on the surface of PBAs through coprecipitation.^143,144^ (ii) In situ ion exchange. Given the fact that the Mn—C≡N—Fe group has a higher solubility constant than Ni-HCF, it is feasible to coat sodium nickel hexacyanoferrate (PBN) on the surface of sodium manganese hexacyanoferrate (PBM), as shown in Figure 11d.^115^ (iii) One-pot synthesis to obtain epitaxial core–shell PBAs because of the unequal formation and stability constants of citrate anion for Ni^2+^ and Mn^2+^. Our group found that Ni^2+^ tends to be released only when Mn^2+^ is completely consumed, resulting in an epitaxial growth of NiPB on the already formed MnPB template.^145^ Benefiting from the highly matched lattice parameters, NiPB exerted a stabilizing counterbalancing strain on the Jahn–Teller-distorted MnN_6_ octahedra. As a result, the MnNiPB-4xcit with an optimized equivalent of Na_3_Cit possessed an appropriate thickness (9% of thickness of MnPB) to imbue the material with phase stability and an ultrahigh capacity retention of 96% after 500 cycles.

##### 4.1.4.3 Conductive Carbon/Polymers

Besides inorganic materials and Ni-HCF, conductive carbons/polymers, including reduced graphene oxide (rGO),^146,147^ polypyrrole (PPY),^140,148^ polydopamine (PDA),^149^ polyaniline (PANI),^150^ and poly(3,4-ethylene dioxythiophene) (PEDOT),^151^ could be applied as the coating layers. The conductive carbon/polymer coating has multiple merits: enhancing the overall electrical conductivity, suppressing the chemical dissolution, preventing unexpected attack of organic electrolyte, reducing the partially oxidized trivalent Fe^3+^/Mn^3+^ by in situ polymerization, and offering additional redox sites to increase the capacity of electrodes by some of the carbonaceous materials, such as PPY and PANI (Figure 11e).

#### 4.1.5 Cationic Doping

Cationic doping (elemental substitution) has been proven to be effective strategy to enhance the capacity, working voltage, and cycling performance for cathode materials in SIBs. The same concept could be used for PBAs with partial substitution of the transition metal coordinated with a N atom (Fe atom coordinated with C is fixed in most cases) or an alkali element. It is confirmed that the species or amount of doping metal has a significant influence on the structural stability and electrochemical behavior of PBAs. Therefore, ingenious regulation of the doping level is crucial for electrochemical performance improvement.

The studies on transition metal doping are mainly focused on DE-PBAs (Fe-HCFs and Mn-HCFs) with high specific capacities but insufficient cycling performances, in which the N-coordinated Fe/Mn could be partially substituted by one or multiple elements to adjust the crystal structure and redox behaviors. For Fe-HCF, the low-spin Fe^LS^ redox at the higher voltage is hard to activate due to the existence of [Fe(CN)_6_] vacancies. Through divalent Ni,^152,153^ Zn,^154^ Cu,^155,156^ or Mn^157^ doping, the capacity contribution of the low spin Fe^LS^ redox could be elevated to decrease the energy barriers of Na^+^ migration. It was found that 3% Ni substitution in Fe-HCF could increase the low spin Fe^LS^ capacity contribution from 28% (27 mA h g^–1^) to 43% (50 mA h g^–1^).^152^ Meanwhile, Yang’s group reported that 11% Zn substitution in FeZn-PB delivered a higher low spin Fe^LS^ capacity of 60.5 mA h g^–1^, which was higher than that of Fe-PB (50 mA h g^–1^) at current density of 20 mA g^–1^.^154^ In addition, it was demonstrated in our group that a sample with 36% Zn substitution shows minor lattice distortion for the simplified and reversible phase transition from cubic to tetragonal.^158^ As for Mn-HCF, a dramatic capacity decay is observed due to the Jahn–Teller distortion of Mn^3+^, and a 10% decrease in Mn–N distances could be detected after a full charge.^159,160^ The effects of doping Fe, Co, and Ni for the cycling and rate performance of Mn-HCF have been investigated in Shibata’s group.^161^ They found that the lifespan and the capacity retention at high rate were significantly increased due to the suppressed Jahn–Teller distortion of Mn^3+^. After that, He and coworkers reported that the phase structure of Mn-HCF would change from rhombohedral to cubic after Sn^4+^ doping, and enhanced capacity retention after 100 cycles at 240 mA g^–1^ was obtained (80.5% vs 54.0% for bare Mn-HCF).^162^ Lately, Jahn–Teller distortion was found by Shao’ group to be repressed through employing Mn vacancies (V_Mn_) in combination with Ni doping.^163^

In order to further enhance the electrochemical performance, multication lattice substitution has been employed.^164,165^ High quality (HQ)-Ni_*x*_Co_1–*x*_[Fe(CN)_6_] PBAs were synthesized through a chelating agent (trisodium citrate)/surfactant (polyvinylpyrrolidone, PVP) coassisted crystallization method with fewer [Fe(CN)_6_] vacancies and water molecules in Han’s group.^166^ As a result, the optimized sample (*x* = 0.3) exhibited a high specific capacity of 145 mA h g^–1^ and prolonged cyclability of 90% capacity retention after 600 cycles. After that, a ternary NiCoFe-PB sample with Co and Fe at the Ni site was prepared by Yang and coworkers. In such a unique electrode material, Co doping enhanced the redox activity of Fe^LS^; meanwhile, Fe doping enhanced the redox activity of Co^HS^.^167^ Additionally, FeCo-*co*-doping could reduce the Na^+^ diffusion resistance within the solid electrolyte interface; thus, an ultralow capacity fading rate of 0.0044% per cycle has been obtained. Moreover, high-entropy PBAs cathode materials with FeMnNiCuCo sharing the N-coordinated M_1_ site for SIB were first reported by Brezesinski’s group.^168^ The equimolar fractions of above five metal cations increased the structural stability and configurational entropy and suppressed the degradation of PBAs cathodes at high voltages. After that, a link between the high-entropy effect and the observed energy storage capabilities of Mn-HCF was established for the first time. By systematic comparison of the structural and chemical properties of high-, medium-, and low-entropy Mn-HCFs, Brezesinki and coworkers concluded that the electrochemical performance enhancement could be ascribed to the entropy-mediated suppression of the Jahn–Teller distortion.^169^ Inspired by the disordered Rubik’s cube, our group synthesized a high-entropy PBA sample as a “proof-of-concept” to demonstrate its application in energy storage devices.^170^ It was revealed that the increased configuration entropy could promote thermal/air stability and afford a zero-strain two-phase (cubic ↔ tetragonal) Na^+^ storage mechanism. As a consequence, the ultralong cycling lifespan over 50 000 cycles (a capacity retention of 79.2%) was achieved.

Owing to the large family of PBAs, the reversible (de)insertion of an alkali ion can be allowed, and K^+^ insertion has been confirmed to exhibit the best reversibility with the highest potential.^171^ Therefore, most researchers are forced on K^+^ doping to improve the structural and electrochemical stability.^172,173^ A low concentration of K^+^ in Na_*x*_K_*y*_FeHCF samples would expand the PBA framework structure and provide a larger cell volume for Na^+^ intercalation.^174^ In addition, K^+^ could be reinserted at 8c sites before Na^+^, providing extra specific capacity and preventing the phase transition and lattice expansion. Then, the synthesis of a series of K_*x*_Na_*y*_MnFe(CN)_6_ (*x* + *y* ≤ 2, KNMF) samples through coprecipitation route was proposed by Qiao’s group, where adjustive sodium citrate was used as sodium resource and organic additive.^175^ KNMF-3 (*x* = 1.59, *y* = 0.25) exhibited better electrochemical performance with good crystallinity, a high Na^+^ content, and nanocubic morphology compared to the sample without K-doping. A facile “potassium-ions assisted” strategy was developed by our group to prepare highly crystallized Fe-based PBAs by controlling the crystal phase orientation.^176^ The optimized product NKPB-3 (Na_0.28_K_1.55_Fe[Fe(CN)_6_]·1.53H_2_O) displayed a stable structure-orientating (220) plane with fewer [Fe(CN)_6_]_4_ vacancies and a lower water content. Attributed to the highly crystal structure and pillar effect of K^+^, the as-obtained electrode delivered a high initial specific capacity of 147.9 mA h g^–1^ and 83.5% capacity retention after 300 cycles.

In order to effectively enhance the doping measures for Fe-HCF materials, it is important to increase the redox activity of Fe^LS^ and the capacity contribution of the low spin Fe^LS^. This can be achieved by incorporating alternative elements, such as Ni, Co, Zn, etc. For Mn-HCF, the key issue is to use the doped elements to limit the Jahn–Teller effect of Mn^3+^ and enhance the structural stability. In this strategy, elements such as Fe, Co, Ni, and Sn were adopted. It was worth noting the introduction of both Co and Ni, as they had dual effects, while taking into account their intake/cost. Furthermore, doping K^+^ into the alkali site is an excellent practice that greatly improves the structural and electrochemical stability. Continuous regulation of the introduced solubility is necessary to achieve the best effect for future development.

### 4.2 Scalable Preparation

**Figure 12:**
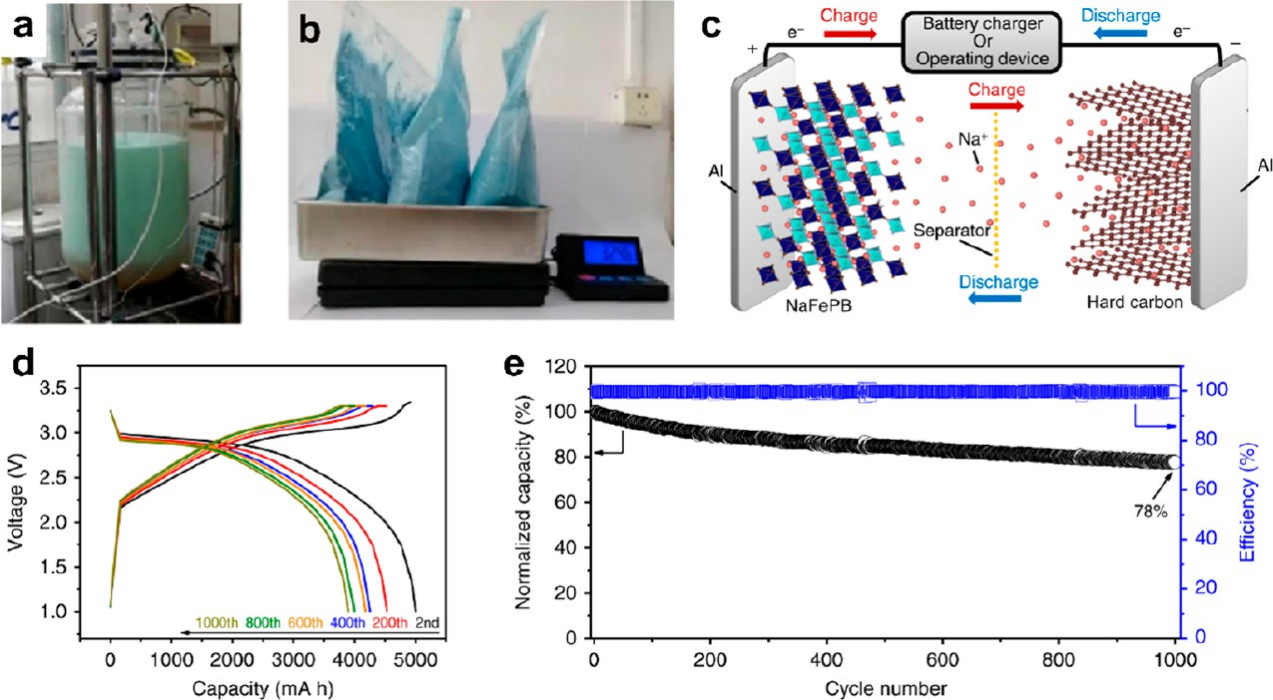
(a and b) Kilogram-grade preparation of MnFeNi-PB. Reproduced with permission from ref (177). Copyright 2022 Elsevier. (c) Working mechanism of a 5 Ah pouch full cell-based Na_2–*x*_FeFe(CN)_6_ cathode and hard carbon anode, (d) charge–discharge curves, and (e) cycling performance of the 5 Ah pouch full cell. Reproduced with permission from ref (179). Copyright 2020 Springer Nature.

Up to now, several groups have begun to prepare PBAs cathode materials on a kilogram-scale. Xie’s group synthesized the Ni and Fe-codoped manganese hexacyanoferrate PB (MnFeNi-PB) via a Na_3_Cit-assisted coprecipitation method.^177^ They realized kilogram-scale MnFeNi-PB using a 100 L reactor, and 3.2 kg of sample could be obtained, as shown in Figure 12a and b. The as-prepared MnFeNi-PB exhibited a long cycle life at room temperature (65.5% capacity after 2000 cycles, 5 C, 1 C = 150 mA g^–1^), 45 °C (83.5% capacity retention, 300 cycles, 1 C), and −20 °C (92.1% capacity retention, 700 cycles, 1 C). After that, they synthesized Mn/Ni binary PBAs in a high precursor salt concentration of 0.5 mol L^–1^ (Mn_0.5_Ni_0.5_-0.5), resulting in a higher yield for mass production.^178^ The result showed that the cycling performance and discharge specific capacity were better than those of the sample prepared at a lower salt concentration. Moreover, Mn_0.5_Ni_0.5_-0.5 displayed excellent cycling performance at overcharge to 4.8 V (91.8% retention) and overdischarge to 1.2 V (89.1% retention) after 300 cycles, demonstrating that it exhibited a satisfactory tolerance for deep charge/discharge. In addition, sodium-rich Na_2–*x*_FeFe(CN)_6_ has been successfully prepared in our group by a scale-up precipitation route with Na_3_Cit as a chelating agent and sodium supplement (yield of 5 kg per 100 L).^179^ It was concluded that Na_3_Cit could play the most important role in crystal growth. With the increase of the Na_3_Cit concentration, the morphology of Na_2–*x*_FeFe(CN)_6_ turned to a single microcube compared to irregular particles at low concentration. Subsequently, A 5 Ah pouch full cell with an as-prepared Na_2–*x*_FeFe(CN)_6_ cathode and hard carbon anode has been assembled, and excellent electrochemical behavior has been achieved (Figure 12c). Additionally, no sodium compensation was added because the sodium atomic ration in this Fe-PBA cathode reached up to 1.73. As shown in Figure 12d and e, an obvious plateau at 2.9 V was observed, and 78% capacity retention could be obtained after 1000 cycles. Such significant work can pave the way for scaled-up preparation of PBAs in the future.

## 5 Outlook and Perspectives

**Figure 13:**
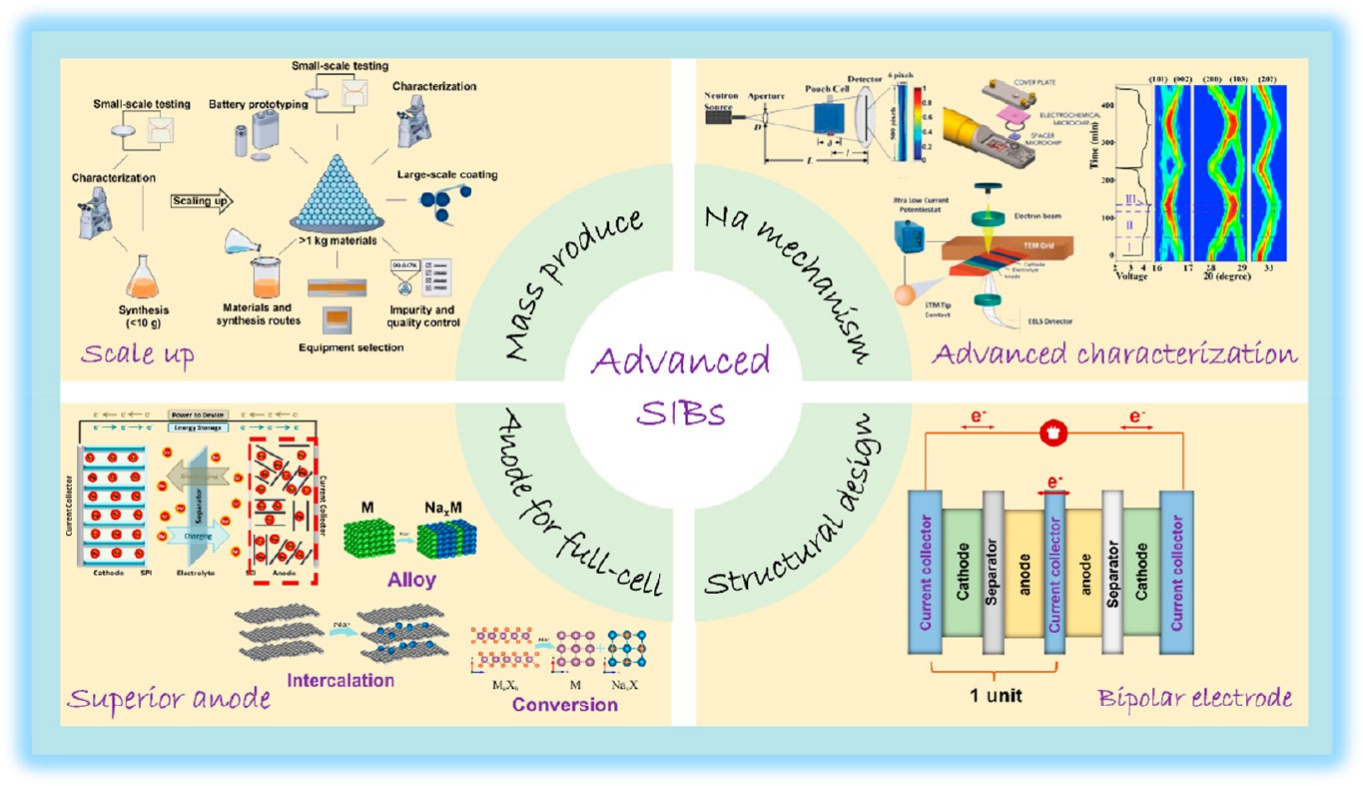
Figure for the development prospects of cathode materials in SIBs. Top left image reproduced with permission from ref^180^. Copyright 2023 Springer Nature. Top right (center) image reproduced with permission from ref (181). Copyright 2019 Wiley-VCH. Far right image reproduced with permission from ref (185). Copyright 2021 Royal Society of Chemistry. Bottom left (center) image reproduced with permission from ref (186). Copyright 2023 American Chemical Society.

In summary, great progress has been made in developing cathode materials for SIBs in recent years in spite of the many challenges remaining. The commonly used cathode materials for SIBs include transition metal oxides, polyanion compounds, and Prussian blue (analogs), which have different physical and chemical properties and electrochemical performance due to their versatile compositions and crystal structures. The transition metal oxides can be simply prepared and demonstrate high specific capacity and good rate capability, but they are prone to collapse during repeated Na insertion/extraction owing to their fragile crystal structures, which is usually resolved by lattice regulation. Polyanionic materials have high working voltages and excellent thermal/cyclic stability due to from their stable crystal structure and strong X–O polar bonds, but they suffer from inferior electronic conductivity. Surface modification and morphology/lattice regulation have been explored to improve the performance of polyanionic cathode. Prussian blue and its analogs have the advantages of low cost, great rate performance, and adjustable working voltages, but the stubborn crystal water causes their chemical and structural instability. In conclusion, there are remaining issues that need to be addressed before the above three categories of cathode materials showcase their grandeur in the field of large-scale energy storage, as discussed in below (Figure 13).

(i) Developing large-scale synthesis techniques: Anode materials, such as hard carbon and silicon, can be easily mass produced with high consistency due to their simple compositions and abundant raw materials, while syntheses of cathodic materials containing complex components and expensive raw materials (e.g., V-, Cu-, and Co-based compositions) usually involve cumbersome synthesis steps, making the mass production of cathode materials highly challenging. Synthesis techniques such as the coprecipitation method and solid-state ball milling can be extended to large scale production with their product yields and batch stability further improved.
(ii) In-depth understanding of the Na storage mechanism: The studies on the in situ structure/component evolution of the electrodes and the genesis of a cathode electrolyte interface (CEI) film during cycling are essential to illustratethe Na storage mechanism and guiding the development of the high performance cathode materials. Therefore, advanced characterization techniques, such as in situ neutron diffraction, in situ X-ray absorption spectroscopy (XAS), and in situ electrochemical monitoring, should be more intensively adopted.
(iii) Seeking matching anodes for full-cell study: The assemble of a Na full cell must consider the matching between the cathode and the anode. High specific capacity is the ultimate pursuit in selecting anode materials, so traditional hard carbon anode materials with limited theoretical capacity cannot meet the needs of high-energy SIBs. Carbon-based anode materials with high specific capacities, such as graphene and carbon nanotubes, have been developed and applied to full cells of SIBs. In addition, alloy–carbon composites and sodium metal anodes are also under consideration, expecting the high expansion of the alloy anode and severe dendritic growth of the Na metal anode will be resolved in the near future.
(iv) Optimizing the construction of SIBs: The bipolar electrode design using inexpensive aluminum as a shared current collector can help achieve efficient recycling of electrode materials, and the absence of alloying reaction between Na and Al is the foundation of this design. Although the construction of SIBs with a bipolar electrode may render a lot of advantages, including higher specific (volumetric) energy density, excellent high-rate performance, and reduced cell resistance, potential electrolyte leakage and subsequent intermixing may cause exhaustive failure of the batteries. The validity and reliability of a variety of battery constructions have yet to be examined.
